# Benthic Bacterial Community Composition in the Oligohaline-Marine Transition of Surface Sediments in the Baltic Sea Based on rRNA Analysis

**DOI:** 10.3389/fmicb.2018.00236

**Published:** 2018-02-19

**Authors:** Julia Klier, Olaf Dellwig, Thomas Leipe, Klaus Jürgens, Daniel P. R. Herlemann

**Affiliations:** ^1^Department of Biological Oceanography, Leibniz Institute for Baltic Sea Research, Rostock, Germany; ^2^Department of Marine Geology, Leibniz Institute for Baltic Sea Research, Rostock, Germany

**Keywords:** brackish microbiology, estuarine ecology, Baltic Sea, bacteriobenthos, bacterial diversity and community composition

## Abstract

Salinity has a strong impact on bacterial community composition such that freshwater bacterial communities are very different from those in seawater. By contrast, little is known about the composition and diversity of the bacterial community in the sediments (bacteriobenthos) at the freshwater-seawater transition (mesohaline conditions). In this study, partial 16S-rRNA sequences were used to investigate the bacterial community at five stations, representing almost freshwater (oligohaline) to marine conditions, in the Baltic Sea. Samples were obtained from the silty, top-layer (0–2.5 cm) sediments with mostly oxygenated conditions. The long water residence time characteristic of the Baltic Sea, was predicted to enable the development of autochthonous bacteriobenthos at mesohaline conditions. Our results showed that, similar to the water column, salinity is a major factor in structuring the bacteriobenthos and that there is no loss of bacterial richness at intermediate salinities. The bacterial communities of marine, mesohaline, and oligohaline sediments differed in terms of the relative rRNA abundances of the major bacterial phyla/classes. At mesohaline conditions typical marine and oligohaline operational taxonomic units (OTUs) were abundant. Putative unique OTUs in mesohaline sediments were present only at low abundances, suggesting that the mesohaline environment consists mainly of marine and oligohaline bacteria with a broad salinity tolerance. Our study provides a first overview of the diversity patterns and composition of bacteria in the sediments along the Baltic Sea salinity gradient as well as new insights into the bacteriobenthos at mesohaline conditions.

## Introduction

Estuaries have strong physico-chemical gradients of salinity, nutrient concentrations, organic matter content, and composition. These gradients reflect the mixing of freshwater and seawater (McLusky and Elliott, 2004) and influence the composition of bacterial communities (Barcina et al., 1997). A global-scale meta-analysis of diverse environments showed that salinity is the major determinant of microbial communities and is more influential than temperature, pH, and other physiochemical factors (Lozupone and Knight, 2007). Accordingly, bacterial community composition differs significantly between marine and freshwater environments. The changes in pelagic microbial communities in response to shifts in salinity have been well-investigated, whereas much less is known about corresponding changes in sediment bacterial communities.

Bacteria drive an essential part of the biogeochemical activity in sediments (Nealson, 1997) and bacterial biomass and taxon richness is usually higher in sediments than in the corresponding water bodies (Zinger et al., 2011). Oxygenated top layer marine sediments are generally dominated by *Deltaproteobacteria* and *Gammaproteobacteria* (Schultz and Urban, 2008; Zinger et al., 2011), and freshwater sediments by *Bacteroidetes* and *Chloroflexi* (Dai et al., 2013; Zhang et al., 2015). The transition between marine and freshwater environments is characterized by extreme osmotic changes, and thus significant physiological adaptations that enable the survival of organisms under mesohaline conditions are required (Walsh et al., 2013). Indeed, similar to the pelagic bacterial community, the benthic bacterial community found at intermediate salinities seems to differ significantly from that of marine and limnic waters (Wang et al., 2012; Vetterli et al., 2015; Lv et al., 2016; Pavloudi et al., 2016).

The Baltic Sea, with its water residence time of >3 years (Reissmann et al., 2009), is well-suited as a model system to investigate autochthonous bacterial communities at intermediate salinities (Herlemann et al., 2011). While benthic invertebrates at salinities of 5–10 in the Baltic Sea are characterized by low diversity (“species minimum concept”; Remane, 1934; Zettler et al., 2014), this is not the case for bacterioplankton (Herlemann et al., 2011, 2016). Several studies have also provided evidence of unique bacterial taxa at intermediate salinities (Crump et al., 2004; Kirchman et al., 2005; Kan et al., 2007; Herlemann et al., 2011, 2016). However, studies of benthic bacterial diversity in the Baltic Sea have been limited to single stations (Edlund et al., 2008; Shubenkova et al., 2010; Vetterli et al., 2015; Reyes et al., 2016), whereas systematic comparisons across the salinity gradient are lacking. Based on previous studies of the water column of the Baltic Sea (Herlemann et al., 2011), we hypothesized in the present study that (1) salinity has a strong impact also on the community composition of sediment bacteria, (2) sediment bacterial richness does not reach a minimum under mesohaline conditions, and (3) unique bacterial taxa/groups are a frequent component of the bacterial communities in mesohaline sediments. To test these hypotheses, we examined surface sediments with comparable physico-chemical features (oxic, silty) at five stations along the salinity gradient of the Baltic Sea.

## Materials and Methods

### Sediment/Porewater Sampling and Geochemical Analyses

**FIGURE 1:**
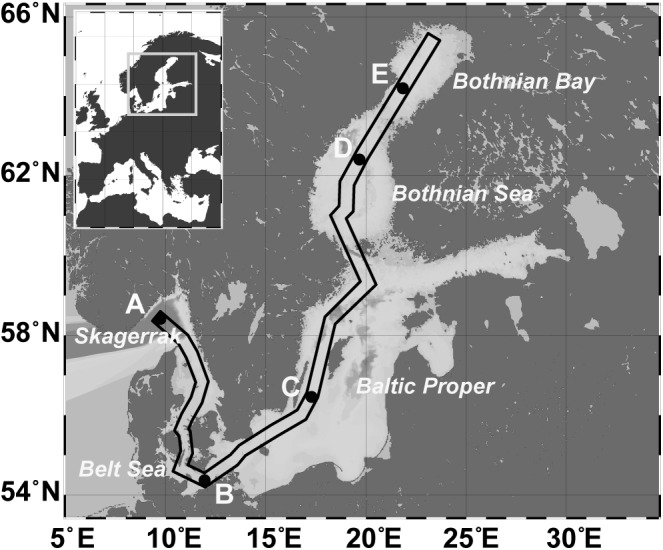
Map of the Baltic Sea, including the sampling stations: A (Skagerrak, salinity 35), B (Belt Sea, salinity 21), C (Baltic Proper, salinity 8), D (Bothnian Sea, salinity 7), E (Bothnian Bay, salinity 4). The box marks the sampling transect used for the profile displayed in Supplementary Figure S1.

Twenty sediment short cores at five stations (A–E) from the Skagerrak to the Bothnian Bay (**Figure 1** and Supplementary Figure S1) were taken using a multicorer device (Oktopus; Kiel, Germany) during a cruise with *R/V* Meteor (M86-1) in November 2011. Temperature, oxygen, and conductivity (salinity) were measured using a SeaBird CTD (SBE911) lowered until it was closely above the sediment. The oxygen concentration in the bottom water directly above the sediment core was determined by Winkler titration (Grasshoff et al., 1983). Based on the salinity of the respective bottom water, station A (Skagerrak, 58° 29.760′N; 9° 35.910′E) was defined as marine (salinity 35.2), station B (Belt Sea; 54° 17.000′N; 11° 34.000′E) as marine-mesohaline (salinity 20.8), stations C (Baltic Proper; 56° 27.000′N; 17° 17.000′E) and D (Bothnian Sea; 59° 21.500′N; 20° 6.000′E) as mesohaline (salinity 8.3 and 7.2), and station E (Bothnian Bay; 64° 12.200′N; 22° 1.700′E) as oligohaline (salinity 3.9) in accordance to the Venice system (Anonymous, 1958). Three sediment cores separated by an approximate distance of 1 m were obtained at each station and analyzed for bacterial community, grain size, water content, and carbon-nitrogen-sulfur (CNS) concentration. A fourth core was used for porewater extraction.

For bacterial community analysis, grain size, water content, and CNS analyses, five upper 0.5-cm slices were prepared: A1 (0–0.5 cm, A2 (0.5–1 cm), B1 (1–1.5 cm), B2 (1.5–2 cm), and C (2–2.5 cm) and transferred to Petri dishes. Cut-off pipette tips were used to remove approximately 5 mL from each petri dish for RNA/DNA extraction. The 5 mL sediment were stored in RNAlater (Qiagen; Hilden, Germany) at -20°C until usage. The remaining sediment was lyophilized and the difference in the weights of the wet vs. the freeze-dried sediment was used to determine the water content. Total carbon (TC), total nitrogen (TN), and total sulfur (TSul) were measured in homogenized subsamples of dried sediment using an elemental analyzer (EA1110 CHN, CE Instruments; Lancashire, United Kingdom), and total inorganic carbon (TIC) using a multi-EA 4000 (Analytik Jena, Germany). For TIC, the sample was treated with 40% H_3_PO_4_ and the released CO_2_ was analyzed using an infrared detector calibrated with a pure CaCO_3_ standard (12.0% TIC). Total organic carbon (TOC) was calculated based on the difference between TC and TIC. Since sulfate concentrations are naturally higher in marine seawater than in oligohaline water, the estimated sea water sulfate (SO_4_^2-^) concentration at the respective salinities (28.2 mM SO_4_^2-^ at a salinity of 35; 16.9 mM SO_4_^2-^ at a salinity of 21; 6.4 mM SO_4_^2-^ at a salinity of 8; 5.6.2 mM SO_4_^2-^ at a salinity of 7; 3.2 mM SO_4_^2-^ at a salinity of 4) was subtracted from TSul to obtain a salinity-corrected value (Sul_red_). Another part of the lyophilized sediment was slurried, analyzed for grain size, and sorting (Q_50_, **Table 1** and Supplementary Table S1) using a laser particle-size-analyzer (CILAS 1180; Orleans, France).

**Table 1:** Physical and chemical characteristics of the sampling sites for the uppermost 2.5 cm of sediment and porewater.

	A	B	C	D	E
**Sampling site**					
Underwater depth [m] (Depth)	548	24	54	48	110
Bottom temperature [°C] (Temp)	6	11	3	6	2
Bottom salinity (Sal)	35	21	8	7	4
Oxygen bottom water [mL/L]	6.1	4.8	3.6	5.6	7.5
SO_4_^2-^ [mM] calculated^∗1^	28.2	16.9	6.4	5.6	3.2
**Sediment characteristics**					
Median grain size [Q_50_]	5	11	12	3–377	10
Sediment type	Fine silt	Middle silt	Middle silt	Silty-sandy	Middle silt
Water content [%]	76	78	54	33	87
Total nitrogen [%] (TN)	0.3 ± 0.0	0.5 ± 0.0	0.3 ± 0.3	0.04 ± 0.0	0.4 ± 0.0
Total organic carbon [%] (TOC)	3.6 ± 0.1	4.0 ± 0.2	2.1 ± 2.0	0.7 ± 0.4	4.0 ± 0.4
C/N_molar_ ratio	11.7 ± 1.7	8.1 ± 0.1	8.7 ± 0.8	8.7 ± 0.8	9.7 ± 0.3
Total sulfur [%] (TSul)	0.3 ± 0	0.8 ± 0.1	0.2 ± 0.2	0	0.1 ± 0
Calculated sulfur [%]^∗2^ (Sul_red_)	0 ± 0	0.5 ± 0.1	0.2 ± 0.2	0	0 ± 0
**Porewater**					
SO_4_^2-^ [mM]	29–30	14–16	N/A	6	N/A
Fe_diss_ [μM]	0–1	0–53	N/A	0	N/A
Mn_diss_ [μM]	0–19	0–20	N/A	0–14	N/A
Si(OH)_3_ [μM]	0–83	37–447	N/A	22–68	N/A
NO_3_^-^ [μM]	7–9	1–2	N/A	2	N/A
TDP [μM]	1–2	2–48	N/A	2–5	N/A
NH_4_^+^ [μM]	2–3	29–160	N/A	0–1	N/A
H_2_S [μM]	0	0	N/A	0	N/A

*A, Skagerrak; B, Belt Sea; C, Baltic Proper, D, Bothnian Sea; E, Bothnian Bay (see **Figure 1**). N/A, not available. ^∗*1*^Calculated SO_*4*_^2-^ concentration based on a concentration of 28.2 mM at a salinity of 35. ^∗2^Sulfur value of the CNS analysis after the calculated SO_*4*_^*2*-^ of ^∗*1*^ was subtracted*.

For the porewater analysis, samples taken at 1-cm distances were extracted onboard using rhizons (rhizosphere; length 5 cm; Ø 2.5 mm, pore size: 0.1 μm) according to Seeberg-Elverfeldt et al. (2005). Porewater H_2_S was measured spectrophotometrically (Specol, Analytik Jena) from 2-mL reaction tubes containing 20 μL of 20 vol% Zn-acetate according to (Cline, 1969). For nitrate, nitrite, and ammonium, 2-mL of porewater was stored frozen in pre-cleaned reaction tubes until measurement of the samples using an autoanalyzer (Quaatro, Seal). For the determination of dissolved Fe, Mn, total dissolved P (TDP), S, and Si, 2 mL of porewater in pre-cleaned reaction tubes was acidified with 20 μL of concentrated HNO_3_ (suprapure) and stored at 4°C until analyzed using inductively coupled plasma-optical emission spectroscopy (ICP-OES, iCAP 6300 Duo, Thermo Fisher Scientific, **Table 1** and Supplementary Table S2). Total S were measured in the porewater by ICP-OES. Since the contribution by reduced S species (e.g., HS-) was negligible total S were used as SO_4_.

### Analysis of Bacterial Community Composition

DNA/RNA was extracted from the sediment samples using a DNA/RNA mini kit (Qiagen; Hilden, Germany) and physically separated in RNA and DNA. Remaining DNA in the RNA sample was digested using the Turbo DNA-free kit (Ambion). The iScript Select cDNA synthesis kit (Bio-Rad Laboratories GmbH; Munich, Germany) was used to transcribe the remaining RNA into cDNA. For station B we used both the RNA and the DNA.DNA from station B and cDNA from all stations were PCR amplified according to the protocol of Herlemann et al. (2011), using 30 cycles to amplify bacterial sequences by the primers Bakt_341F and Bakt_805R (Herlemann et al., 2011). The amplicons were purified using Agencourt© AMPure^®^ XP (Becker Coulter) and sequenced at Eurofins MWG Operon using 454 pyrosequencing based on Roche GS FLX titanium series chemistry. The resulting sequences were quality checked by RDPpyro (Cole et al., 2013) according to the following settings: maximum number of *N* = 0, minimum sequence length = 150, and minimum exponential *Q*-score = 20. They were then evaluated using the SILVA next -generation sequencing (NGS) pipeline (Glöckner et al., 2017) based on SILVA release version 115 (Pruesse et al., 2007). SILVA NGS performs additional quality checks according to the SINA-based alignments (Pruesse et al., 2012) with a curated seed database in which PCR artifacts or non-SSU reads are excluded. The longest read serves as a reference for the taxonomic classification in a BLAST (version 2.2.28+) search against the SILVA SSURef dataset. The classification of the reference sequence of a cluster (98% sequence identity) is then mapped to all members of the respective cluster and to their replicates. Best BLAST hits were only accepted if they had a (sequence identity + alignment coverage)/2 ≥ 93 or otherwise defined as unclassified. SILVA NGS classified a total of 525,558 reads (0.7% were rejected by the quality control). De-replication yielded 173,001 sequence clusters that were assigned based on their rank-taxonomy to 1,062 bacterial taxa representing on average bacterial genera (operational taxonomic units: OTUs). Sequences present only once in the dataset (singletons) were excluded, as were those assigned to chloroplasts and *Archaea* since the primer set employed in the analysis has only a very limited coverage of these groups. The raw reads were deposited at the European Nucleotide Archive under study accession PRJEB7250, and sample accessions ERS542654- ERS542736.

To complement the SILVA-NGS-based, taxonomic-rank-based assignments, the most abundant sequences affiliated with a defined bacterial genus in SILVA NGS were analyzed using the ARB software suite (Ludwig et al., 2004). The closest phylogenetic relative in the database was defined by adding the most abundant sequences to the Silva_128_NR tree using the quick-add tool provided in ARB. This procedure adds short sequences without changing the global tree topology and therefore well estimates their phylogenetic placement. The related full-length sequences were used to calculate a base tree and the most abundant short sequences from this study, were again added. The program *seqenv* (Sinclair et al., 2016) was used to annotate the most abundant read from an OTU to an environmental term based on the Environmental Ontology (*EnvO*) vocabulary (Buttigieg et al., 2016).

### Statistical Analysis

Richness was estimated using Explicet (Robertson et al., 2013) based on a bootstrapped rarefaction (rarefaction point at 12,833 reads). A Kruskal-Wallis test and a *post hoc* Tukey’s pairwise test was used to calculate significant differences between the number of OTUs in the samples. Variations in bacterial community structure were characterized in a principle coordinates analysis (PCoA) using the Bray–Curtis dissimilarity in the “vegan” community ecology package of R Studio (Oksanen et al., 2013) based on read abundances normalized to total reads per sample. The PCoAs were carried out using OTUs with an abundance of >0.9% of the total reads. For clarity, the relative abundance cut-off level for phyla/classes was 1.8% of the rRNA reads for each individual OTU in the examined class or phylum. Bacterial communities were correlated according to the environmental parameters using the *envfit* program included in “vegan”. Only the environmental parameters available for all stations were used; the bottom oxygen concentration was excluded because it can vary significantly in the different sediment layers. A linear discriminant analysis effect size (LEfSe) analysis (Segata et al., 2011) was used to identify bacterial groups whose relative abundance differed significantly between samples. For this purpose, the default setting with the multi-class analysis the “One against all” was used. OTUs identified in the LEfSe as significantly enriched were defined as indicator OTUs.

## Results

### Sediment Characteristics

Sediment samples were collected from sites with a salinity range of 4–35 (**Table 1** and Supplementary Figure S1). The water temperature ranged from 2°C to 11°C and was lowest in the Bothnian Bay (station E) and highest in the Belt Sea (station B). Samples were chosen from sites where bioturbation was minimal (no large wormholes or coverings); the fluffy layer was removed. With the exception of station D, the median grain size (Q_50_) was in the range of 5–12, compatible with fine silt-middle silt (**Table 1**). The mixed (sandy-silty) sediment at station D had the lowest TC and TN contents. The C/N ratio was comparable between the stations (**Table 1**). After SO_4_^2-^ correction, only station B contained measurable Sul_red_ (0.5%, **Table 1**). At station C, Sul_red_ was within the range of variation in the measurements. Porewater H_2_S concentrations throughout the upper 2.5 cm were below the detection limit (>1 μM) of the method used, but at station B they were increased at a sediment depth > 4.5 cm (Supplementary Table S2). Station B samples had the highest porewater concentrations of Fe_diss_, TDP Si(OH)_3_, and NH_4_^+^, and station A samples the highest concentration of nitrate and SO_4_^2-^ (**Table 1**).

### Benthic Bacterial Diversity and the Impact of Abiotic Factors

**FIGURE 2:**
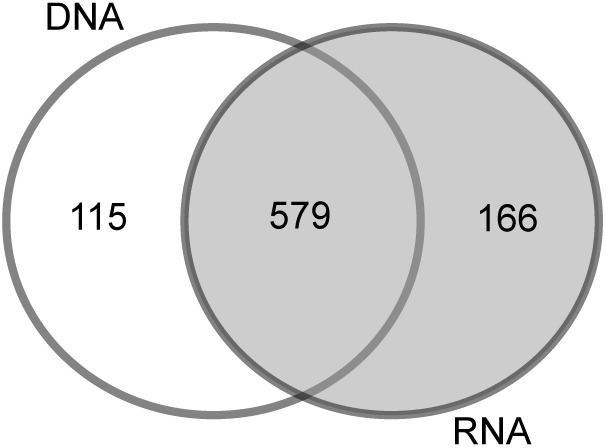
Venn diagram of the OTU overlaps between the DNA- and RNA-based samples of station B.

To determine the impact of the template used for the analysis (RNA or DNA), we compared the bacterial community of station B based on the RNA vs. the DNA template. In addition to the 579 OTUs (68%) that were similar in the DNA and RNA analyses, 115 OTUs (13%) were specifically found in the DNA-based analysis and 166 (19%) in the RNA-based analysis (**Figure 2**). The PCoA of OTU composition and abundance showed a clear separation of the DNA- and RNA-derived bacterial communities (Supplementary Figure S2a). Although the 20 most abundant OTUs were comparable between the samples, they differed in their relative abundances (Supplementary Figure S2b). This was especially the case for “Acidiferrobacter OTU*,” “*Sva0081 OTU,” and *“*Thiotrichaceae OTU,” the abundances of which were higher in the RNA than in the DNA samples (Supplementary Figure S2b). We therefore used the RNA-based samples for further analyses as it provides also a picture of the more active part of the community (and the protein biosynthesis potential; Blazewicz et al., 2013).

**FIGURE 3:**
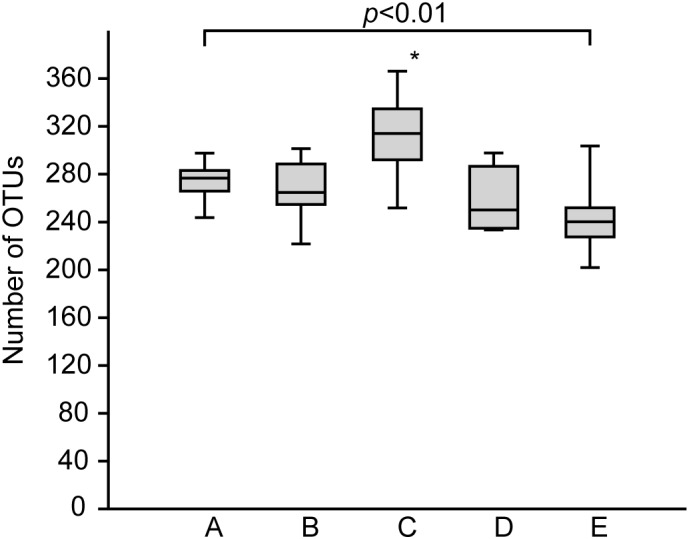
Boxplots showing the rarefied and bootstrapped number of operational taxonomic units (OTUs) at the different sampling stations. The boxplots show the 25–75 percent quartiles; the median is indicated by the horizontal line inside the box. A (Skagerrak, salinity 35, *n* = 14), B (Belt Sea, salinity 21, *n* = 15), C (Baltic Proper, salinity 8, *n* = 15), D (Bothnian Sea, salinity 7, *n* = 9), E (Bothnian Sea, salinity 4, *n* = 13). See also **Figure 1** for an overview. The asterisk indicates significant differences (Tukey *p* < 0.01) between station C and stations A, B, D, and E. Station E was significantly different from station A.

**FIGURE 4:**
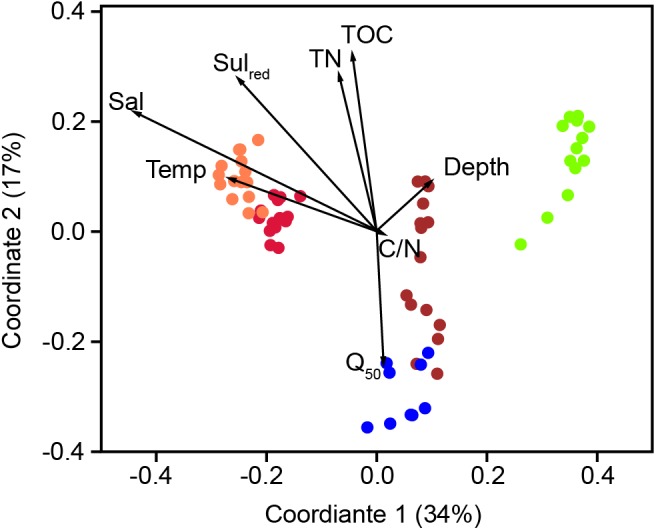
Principle coordinate analysis using the Bray–Curtis similarity index of the bacterial community composition based on OTUs with a RNA abundance of > 0.9% of the total reads. A (red, salinity 35); B (orange, salinity 21); C (brown, salinity 8); D (blue, salinity 7) mesohaline; E (green, salinity 4). The vectors were added *post hoc* and are based on the *envfit* analysis. Temp, temperature; Sal, bottom salinity; Depth, underwater depth; Q_50_, median grain size; TN, total nitrogen; C/N, molecular ratio of total organic carbon/total nitrogen; Sul_red_, calculated sulfur concentration.

The bacterial communities in the sediments of stations A–E were compared with respect to their transcribed 16S rRNA fragments. Bacterial richness (α-diversity; **Figure 3**) was significantly higher at the mesohaline station (C) than at the other stations (Tukey test, *p* < 0.01, Supplementary Table S3) and significantly lower at the oligohaline station (E) than at station A. The bacterial community of the different sediment horizons and replicate cores from each station clustered closely together in the PCoA plot (**Figure 4**). While the marine and marine-brackish stations (A, B; salinity 32, red dots and 21, orange dots) overlapped slightly, they were clearly separated from stations C, D (salinity 8, brown dots and 7, blue dots), and E (salinity 4, green dots). The bacterial communities of samples from the brackish water stations (C and D, salinity 8 and 7) were distinct from those of station E (salinity 4) samples and they differed from each other on the second coordinate. As indicated by the *envfit* results, the main environmental factors separating the total bacterial community were salinity (*r*^2^ = 0.50) and Sul_red_ (*r*^2^ = 0.40) (**Table 2**).

**Table 2:** Results of *envfit* analyses (*r*^2^) testing the correlation between environmental characteristics and the composition of the microbiota determined in the principal coordinate analysis plot (**Figure 4** and Supplementary Figures S3–S7).

	All taxa	*α-proteobacteria*	*δ-proteobacteria*	*γ-proteobacteria*	*Bacteroidetes*	*Chloroflexi*
Q_50_	0.25***	**0.37^∗∗∗^**	0.12*	0.18**	0.17***	0.31***
TN	0.30***	0.09*	0.10*	0.32***	0.22*	0.13*
Sul_red_	0.40***	0.07	0.26***	0.52***	0.30***	0.33***
TOC	0.33***	0.10*	0.11*	0.34***	0.24	0.08
C/N_molar_ ratio	0.01	0.07	0.04	0.01	0.03	0.04
Temp^1^	0.31***	0.09	0.25***	0.42***	0.24**	0.21***
Sal^1^	**0.50^∗∗∗^**	**0.18^∗∗∗^**	**0.36^∗∗∗^**	**0.60^∗∗∗^**	**0.42^∗∗∗^**	**0.39^∗∗∗^**
Depth^2^	0.14**	0.16**	0.21***	0.14**	0.09***	0.19**

*The estimated seawater SO_*4*_^*2*-^ concentration (28.2 mM at a salinity of 35) was subtracted from the total sulfur (Sul) to obtain a salinity-corrected Sul_*red*_ value. (^∗^*p* < 0.05; ^∗∗^*p* < 0.01; ^∗∗∗^*p* < 0.001). Temp, temperature; Sal, bottom salinity, Depth, underwater depth; Q_*50*_, median grain size; TN, total nitrogen; C/N, molecular ratio of total organic carbon/total nitrogen; Sul_*red*_, calculated sulfur concentration. The highest values are indicated in bold. ^*1*^Measured above the sediment. ^*2*^Underwater depth*.

### Taxonomic Groups Identified at Different Salinities Based on 16S rRNA Abundance

**FIGURE 5:**
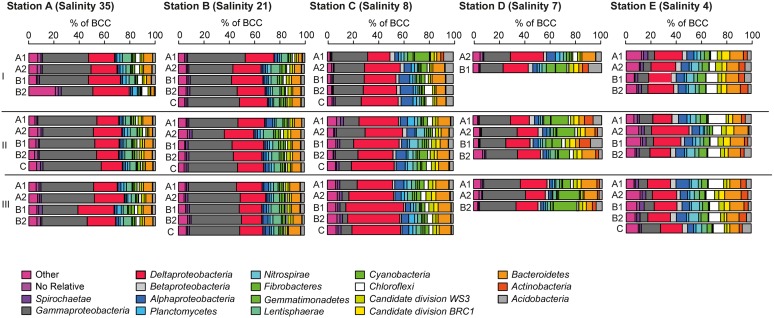
Bacterial community composition at the phylum/class level at **A** (salinity 35); **B** (salinity 21); **C** (salinity 8); (**D**, salinity 7) mesohaline; (**E**, salinity 4) (see **Figure 1**) and in sediment cores (I–III) and sediment horizons (A1–C). The relative abundance cut-off level for abundant phyla/classes on rRNA level were 1.8% in the examined class or phylum.

At the five investigated sites, *Alphaproteobacteria*, *Bacteroidetes*, *Chloroflexi*, *Deltaproteobacteria*, *Gammaproteobacteria, Cyanobacteria*, and *Acidobacteria* were the most dominant phyla/classes on rRNA basis, constituting more than two-thirds of the total reads. In line with the results of the PCoA, characteristic changes in the relative abundances of some of these phyla/classes along the salinity gradient were detected (**Figure 5**).

**FIGURE 6:**
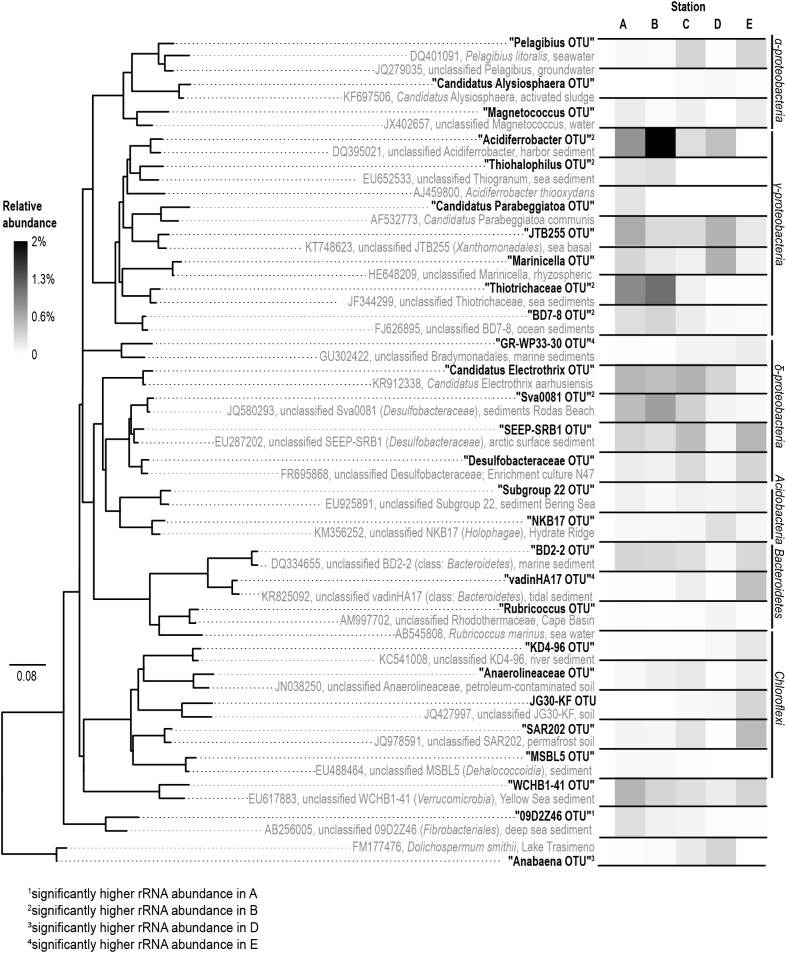
Phylogeny **(left)** and abundance **(right)** of the rRNA based abundant OTUs. The phylogenetic tree was constructed using full-length sequences (gray) representing close relatives of the short-sequence 16S rRNA obtained in our study (bold) as represented by the most abundant rRNA sequences of an OTU. A (salinity 35); B (salinity 21); C (salinity 8); D (salinity 7); E (salinity 4).

*Gammaproteobacteria* was the most abundant class in the dataset. According to *envfit*, the strongest correlation of *Gammaproteobacteria* was with salinity (*r*^2^ = 0.60), and Sul_red_ (*r*^2^ = 0.40, **Table 2**). The relative abundance of *Gammaproteobacteria* in Baltic Sea sediments decreased from the marine (40%) to the oligohaline (7%) stations (**Figure 5**). The most dominant OTU was assigned to the genus *Acidiferrobacter*, which was highly abundant at all stations except E (2.0–0.3%, **Figure 6** and Supplementary Figure S3). The LeFSe analysis indicated that “Acidiferrobacter OTU,” “BD7-8 OTU,” “Thiohalophilus OTU,” and “Thiotrichaeceae OTU” were indicator taxa for station B (**Figure 6** and Supplementary Table S4). *Deltaproteobacteria* were almost as abundant as *Gammaproteobacteria* (15–39%) but its highest rRNA abundance was at station C (**Figure 5**). According to the *envfit* analysis, salinity (*r*^2^ = 0.36) and Sul_red_ (*r*^2^ = 0.26) also had the strongest influence on deltaproteobacterial community composition (**Table 2**). The most abundant OTU was assigned to the *Candidatus* Electrothrix (Trojan et al., 2016; Supplementary Figure S4), abundant at the marine station of the Baltic Sea but also at the other stations with the exception of station E (0.6–0.3%; **Figure 6**). By contrast, the abundance of “GR-WP33-30 OTU” (Order: *Bradymonadales*) was significantly higher at oligohaline station E (0.7%; **Figure 6**). The sequences related to the most abundant sequence of “GR-WP33-30 OTU” were, however, derived from marine sediments (NCBI acc. no. GU3024223). “Sva0081 OTU” was identified as an indicator taxon at station B (0.8% relative abundance) and a related sequence was previously derived from beach sediments (NCBI acc. no. JQ580293, **Figure 6**). Among the *Proteobacteria*, the lowest abundance was that of *Alphaproteobacteria* (1–11% relative abundance) and the changes in the response to salinity were not as strong in this class as in the other abundant proteobacterial classes (**Figure 5**). Rather, according to the *envfit* analysis, among the measured environmental parameters sediment grain size (Q_50_) had the greatest impact on the distribution of *Alphaproteobacteria* (*r*^2^ = 0.37). The most abundant OTU was “Pelagibius OTU,” the abundance of which was highest at stations C and E (0.3% each; **Figure 6** and Supplementary Figure S5). However, this OTU was almost absent at stations D and B (**Figure 6**). The most abundant sequences of the “Pelagibius OTU” were related to *Pelagibius litoralis* and sequences retrieved from freshwater (NCBI acc. no. JQ279035, **Figure 6**).

The relative abundance of *Bacteroidetes* increased slightly from the marine (5–9% relative abundance) to the oligohaline (7–13%) stations (**Figure 5**). The highest values in *envfit* were those of salinity (*r*^2^ = 0.42) and Sul_red_ (*r*^2^ = 0.30, **Table 2**). The most abundant OTU (“BD2-2 OTU”) was present at high relative abundances (0.3%) in all samples except those of station D (**Figure 6** and Supplementary Figure S6). At oligohaline station E, “VadinHA17 OTU” had the highest relative abundances (0.5%), and was also identified as an indicator OTU. Related sequences were previously detected in samples from tidal zones (NCBI acc. no. KR741413, **Figure 6**). Both “VadinHA17 OTU” and “BD2-2 OTU” were absent at station D whereas the “Rubricoccus OTU” was relatively abundant (0.1% relative abundance) at that station and accounted for one quarter of all the *Bacteroidetes* reads. Phylogenetic analysis suggested that the sequences were only distantly related to *Rubricoccus marinus* and formed a separate cluster related to sequences from deep-sea sediments (NCBI acc. no. AM997702, **Figure 6**). The abundance of *Chloroflexi* also increased from the marine (1–2% relative abundance) to the oligohaline (5–14%) stations (**Figure 5**), consistent with the highest *envfit* values for salinity (*r*^2^ = 0.39) and Sul_red_ (*r*^2^ = 0.31; **Table 2**). The most abundant OTU was “SAR202 OTU” (0.5% relative abundance at station E, Supplementary Figure S7), related to sequences from permafrost soil samples (NCBI acc. no. JQ978591).

Besides the relatively diverse and abundant bacterial phyla/classes described above, single OTUs from the *Lentisphaerae, Fusobacter, Chlorobi* and *Fibrobacteres* were identified as indicator taxa and present in high abundance (**Figure 6** and Supplementary Table S4). The *Fibrobacteres* lineage “09D2Z46” was assigned to station A (marine, salinity 35) and related sequences were previously isolated from deep-sea sediments (NCBI acc. no. JN495294). Besides the indicator taxa for specific stations the “WCHB1-41 OTU” comprised almost 2% of the total reads in our dataset. This unclassified lineage has been assigned to the phylum *Verrucomicrobia* and showed no preference for specific salinity conditions.

## Discussion

This study characterized the benthic bacterial community across a salinity gradient in the Baltic Sea, using samples obtained at stations representing stable marine, mesohaline, and oligohaline conditions. The results supported our first hypothesis, that salinity is a major determinant of the sediment bacterial community, as well as the second, that the diversity of bacterial communities at intermediate salinities is comparable to that in marine sediments. However, the third hypothesis, postulating unique bacteria at mesohaline conditions, was only partially supported because while unique OTUs were indeed detected at this salinity range they were only of low abundance.

Sequencing biases, adequate taxonomic resolution, and other potential sources of error (e.g., PCR error, sequencing error, sequencing depth, and bioinformatics analysis) are common concerns in studies relying on NGS approaches (Huber et al., 2009; Quince et al., 2009; Huse et al., 2010; Zhou et al., 2011). We have minimized potential biases by using the standardized SILVA NGS approach for data analysis (Glöckner et al., 2017) in combination with the most universal bacterial PCR primer (Klindworth et al., 2012). Previous studies showed that, especially for sediment samples, DNA-based analysis may also include extracellular DNA bound to sediment particles (Naviaux et al., 2005) as well as inactive microbial communities settled from the water column (Stoeck et al., 2007). In our study, most of the OTUs were present in the DNA- and RNA-generated libraries (579 OTUs) and only about 150 low-abundance OTUs were specifically found in a respective fraction (**Figure 2**). The congruent results of the DNA- and RNA-based analysis suggest that the sequencing effort applied in this study included only a small amount of dissolved DNA or inactive bacteria. Our decision to nonetheless use RNA for most of the analyses was based on our expectation that it would represent the more active (=higher protein biosynthesis potential; Blazewicz et al., 2013) portion of the bacterial community.

### Bacterial Richness

In accordance to a recent study in a Mediterranean lagoon (Pavloudi et al., 2017) the bacterial richness was significantly higher, at the mesohaline condition (station C) (**Figure 3**). However, variability within mesohaline conditions was apparent, probably driven by the abiotic properties of the sediments. For example, at station D the number of OTUs was significantly lower even though the salinity (salinity 7) was close to that at station C (salinity 8). Station D was characterized by a sandy-silty sediment, which is typically lower in TN and TC. Therefore, factors other than salinity, including sediment type, temperature, carbon and nutrient contents, may influence bacterial diversity in the sediments. Similar hypotheses were proposed for pelagic bacterial communities in the Baltic Sea (Herlemann et al., 2016) and for the bacterial communities of tidal sediments (Lv et al., 2016). Since these factors strongly change, including between seasons, the observed peak in richness in the mesohaline sample at station C may have reflected a local effect. Studies comparing the impact of different sediment types and temperatures are needed to understand the changes in bacterial richness in sediments in more detail. Nevertheless, a significant drop in bacterial richness at intermediate salinities can be excluded when comparing stations with comparable physiochemical characteristics (stations A, B, C, E). This is in contrast to the Remane species minimum concept, which proposes a salinity-induced species minimum at intermediate salinities for macrozoobenthos in the Baltic Sea (Remane, 1934; Zettler et al., 2014). Deviations from the species minimum concept were already shown for pelagic unicellular organisms in the Baltic Sea (Telesh et al., 2011; Herlemann et al., 2016). Telesh et al. (2011) suggested distinguishing between the effects of salinity on large benthic versus small motile pelagic organisms because of their different lifestyles. Our study extends this suggestion to include benthic bacteria (bacteriobenthos).

### Environmental Factors Regulating Bacterial Community Composition

In accordance with previous studies in tidal estuaries (Lv et al., 2016) and lagoons (Pavloudi et al., 2016), salinity was the major factor in structuring the bacteriobenthos in our study (**Table 2**). However, the stations along the salinity gradient (A–E) did not follow exactly the change in salinity in the first PCo (**Figure 4**). On the first coordinate, station B (salinity of 21) preceded station A (salinity of 35), presumably due to low oxygen conditions in the deepest layer of station B. This layer contained high concentrations of NH_4_^+^, TDP, and dissolved Fe_diss_ as well as low concentration of NO_3_^-^. The overlaying bottom water contained significant amounts of oxygen and low concentrations of Fe_diss_ (**Table 1** and Supplementary Table S1). These are also favorable conditions for iron-oxidizing bacteria such as *Acidiferrobacter*, which were dominant at this station. Previous studies of the pelagic salinity gradient of the Baltic Sea revealed typical marine bacteria at salinities of 10–35 (Herlemann et al., 2016). Hence, we hypothesize that the clustering and overlap of stations A and B in the PCoA reflected the presence of typical marine bacteria at both stations and that the specific chemical conditions (especially Sul_red_) at station B caused its shift in the PCoA. The second coordinate separated bacterial community mostly based on Q_50_, TOC, and TN (**Figure 4** and **Table 1**). These are sediment parameters that distinguish sandy from silty sediment and their influence on bacterial community was previously reported (Zheng et al., 2014). In our study, different sediment types could be largely circumvented, with the exception of station D, which was separated from station C on the second coordinate despite comparable salinities (salinity 7 and 8, respectively).

### Bacterial Community Composition at Mesohaline Conditions

The OTUs that were significantly enriched at the mesohaline stations C and D belonged either to *Cyanobacteria* or had an overall low abundance (<0.25%). *Cyanobacteria* were in general not further investigated in this study since the short 16S rRNA fragments used in this study provide only very limited information about the phylogenetic assignment (Haverkamp et al., 2009). In the absence of clear indicator taxa for the mesohaline stations, we concluded that the abundant mesohaline bacteria were also present at marine and oligohaline conditions. This was supported by the abundance patterns of the OTUs, since the maximum abundances of most OTUs occurred either at marine (stations A, B; salinity 35 and 21) or oligohaline (station E; salinity 4) conditions (**Figure 6**). The oligohaline conditions examined in this study may not have represented real freshwater conditions and the mesohaline bacteria may have been too similar to the oligohaline bacteria, impeding a clear distinction with the bacteria of the freshwater environment. However, the bacterial community of freshwater environments can differ significantly (Newton et al., 2011) therefore are typical freshwater bacterial community difficult to define. In addition, in our analysis only the *EnvO* terms “marine” and “lake” were abundantly identified whereas the term “estuarine” or “brackish water” was lacking (Supplementary Table S5). This suggests that the bacterial community of the mesohaline sediment consisted of marine and oligohaline OTUs able to tolerate changes in salinity. Typical marine bacteria, especially salinity generalists, can be recruited from freshwater lake sediments (Comte et al., 2016). In addition, genes conferring salinity tolerance have been detected in glacial Baltic Sea freshwater sediment bacterial communities (Marshall et al., 2017). These findings support the hypothesis that many species of sediment bacteria have a wide salinity tolerance that enables them to populate environments with intermediate salinities.

For bacterioplankton, a short water residence time, as is typical in most estuaries, results in a simple mixture of seawater and freshwater bacteria (Crump et al., 2004). However, in the Baltic Sea, with its long water residence time, several bacterial lineages, including SAR11-IIIa (Herlemann et al., 2014) and *Spartobacteria* (Herlemann et al., 2013), are highly abundant at mesohaline conditions, supporting the presence of an adapted mesohaline bacterioplankton community (Crump et al., 2004; Kan et al., 2007). Sediment bacteria are, in contrast to bacterioplankton, more dependent on their local environment and seldom transported by water currents. Therefore, they are directly exposed to changes in salinity just as bacterioplankton during fast water mixing and have therefore a different strategy for adaption than bacterioplankton. However, this hypothesis remains to be validated by additional research into the salinity adaptations of bacteriobenthos.

### Changes in the Taxonomic Composition

The phylogenetic assignment of the OTUs supported the hypothesis that the mesohaline bacteriobenthos is a mixture of marine and freshwater OTUs. Typical marine gammaproteobacterial and deltaproteobacterial OTUs as well as typical freshwater chloroflexal and bacteroidetal OTUs were abundantly present at mesohaline conditions (**Figure 5**). An example of the large salinity tolerance by some bacterial taxa detected in our study was the gammaproteobacterial “Acidiferrobacter OTU.” *Acidiferrobacter* is found in other coastal and tidal sediments (Lenk et al., 2011; Dyksma et al., 2016) where strong changes in salinity can occur. The second most abundant gammaproteobacterium, “Thiotrichaceae OTU,” was present at stations A–C. The abundance pattern together with the phylogenetic and the *seqenv* analyses (Supplementary Table S5) suggested that these OTUs are typical marine OTUs capable of populating mesohaline conditions. Deltaproteobacterial “Sva0081 OTU” and “*Candidatus* Electrothrix OTU” were present at all salinities with the exception of the oligohaline samples of station E (**Figure 6**). Their *EnvO* terms (Supplementary Table S5) and abundance pattern suggested a marine origin. In contrast to those OTUs, the increased abundance of “GR-WP33-58 OTU” from mesohaline to oligohaline conditions was consistent with an oligohaline origin and a tolerance of mesohaline conditions. Similarly, the “Pelagibius OTU,” with related sequences mainly derived from freshwater environments, had the highest abundances at the mesohaline-oligohaline stations, with the exception of station D (**Figure 6**). This result indicates that this is a typical oligohaline OTU well adapted to mesohaline conditions.

*Bacteroidetes* and *Chloroflexi* are cosmopolitans, colonizing marine and freshwater sediments (Llobet-Brossa et al., 1998; Thomas et al., 2011). They have been found in sediments within a broad salinity range and are often involved in the degradation of plant-derived organic matter or other carbohydrates (Hug et al., 2013). Their aerobic lifestyle and capacity to degrade complex organic matter (Holmes, 2006; Reichenbach, 2006) commit these groups to colonize the surface of the sediment, where they can profit from the sinking of relatively recalcitrant organic material. Accordingly, the most abundant OTUs from *Bacteroidetes* and *Chloroflexi* were less abundant in sandy sediment (D), where the TOC concentration is lower. “VadinHa17,” for example, is connected with the use of recalcitrant residual carbon (Baldwin et al., 2015) and was most abundant at oligohaline station E. At station E the concentration of recalcitrant terrigenous carbon is also higher due to the strong terrestrial influence in this area (Herlemann et al., 2017; Seidel et al., 2017). Similarly, the typical *EnvO* term “wetland” suggested its terrigenous origin (Supplementary Table S5). *Chloroflexi* were also detected in all samples, but their abundances were highest at oligohaline station E. This included “SAR202 OTU,” which was present at all stations except station D (**Figure 6**). The higher abundance of this OTU at oligohaline conditions indicates its preference for silty sediment and oligohaline conditions, consistent with the *EnvO* term “permafrost” and “soil” (Supplementary Table S5). An exception to this trend of the *Chloroflexi* was the “MSBL5 OTU,” which was mainly detected in the marine Baltic Sea sediment but at lower abundances at mesohaline salinities (**Figure 6**). This unclassified phylogenetic lineage was previously detected in samples from saline brine lakes and other marine habitats (Pachiadaki et al., 2014) and is therefore presumably adapted to saline conditions.

## Conclusion

This study provided a first overview of the sediment bacterial community composition along the Baltic Sea salinity gradient. Salinity was identified as the most important factor in structuring the bacterial community of oxic, mostly silty sediments. However, differences in sulfur and grain size also resulted in the presence of specific OTUs. Because bacterial richness did not decrease at salinities < 30, other factors, such as sediment type, temperature, carbon and nutrient contents, likely influence bacterial richness. The bacterial community under the mesohaline condition clearly differed from the marine or oligohaline bacterial community. However, both the abundance pattern and the phylogenetic affiliation of the OTUs indicated that either typically oligohaline or typically marine OTUs populated the mesohaline conditions. The OTUs that occurred exclusively at mesohaline conditions were found only in very low abundances. Overall, our results suggest that several typical marine and oligohaline sediment bacteria have been able to populate the mesohaline environment based on their wide salinity tolerance. More research about the ecology of the abundant OTUs at mesohaline conditions is needed to confirm this hypothesis and to elucidate the physiological mechanism that provides the basis for this salinity tolerance.

## Author Contributions

JK, KJ, and DH conceived and designed the study; JK, OD, TL, and DH took the samples and performed analysis; JK and DH analyzed the data. All authors contributed to the writing of the manuscript.

## Conflict of Interest Statement

The authors declare that the research was conducted in the absence of any commercial or financial relationships that could be construed as a potential conflict of interest.

## References

[B1] Anonymous J. (1958). The Venice system for the classification of marine waters according to salinity. *Limnol. Oceanogr.* 3 346–347. 10.4319/lo.1958.3.3.0346

[B2] Baldwin S. A. Khoshnoodi M. Rezadehbashi M. Taupp M. Hallam S. Mattes A. (2015). The microbial community of a passive biochemical reactor treating arsenic, zinc, and sulfate-rich seepage. *Front. Bioeng. Biotechnol.* 3:27. 10.3389/fbioe.2015.00027 25798439 PMC4351619

[B3] Barcina I. Lebaron P. Vives-Rego J. (1997). Survival of allochthonous bacteria in aquatic systems: a biological approach. *FEMS Microbiol. Ecol.* 23 1–9. 10.1111/j.1574-6941.1997.tb00385.x

[B4] Blazewicz S. J. Barnard R. L. Daly R. A. Firestone M. K. (2013). Evaluating rRNA as an indicator of microbial activity in environmental communities: limitations and uses. *ISME J.* 7 2061–2068. 10.1038/ismej.2013.102 23823491 PMC3806256

[B5] Buttigieg P. L. Pafilis E. Lewis S. E. Schildhauer M. P. Walls R. L. Mungall C. J. (2016). The environment ontology in 2016: bridging domains with increased scope, semantic density, and interoperation. *J. Biomed. Semant.* 7:57. 10.1186/s13326-016-0097-6 27664130 PMC5035502

[B6] Cline J. D. (1969). Spectrophotometric determination of hydrogen sulfide in natural waters. *Limnol. Oceanogr.* 14 454–458. 10.4319/lo.1969.14.3.0454

[B7] Cole J. R. Wang Q. Fish J. A. Chai B. Mcgarrell D. M. Sun Y. (2013). Ribosomal database project: data and tools for high throughput rRNA analysis. *Nucleic Acids Res.* 42 D633–D642. 10.1093/nar/gkt1244 24288368 PMC3965039

[B8] Comte L. Cucherousset J. Boulêtreau S. Olden J. D. (2016). Resource partitioning and functional diversity of worldwide freshwater fish communities. *Ecosphere* 7:e01356 10.1002/ecs2.1356

[B9] Crump B. C. Hopkinson C. S. Sogin M. L. Hobbie J. E. (2004). Microbial biogeography along an estuarine salinity gradient: combined influences of bacterial growth and residence time. *Appl. Environ. Microbiol.* 70 1494–1505. 10.1128/AEM.70.3.1494-1505.2004 15006771 PMC365029

[B10] Dai J. Tang X. Gao G. Chen D. Shao K. Cai X. (2013). Effects of salinity and nutrients on sedimentary bacterial communities in oligosaline Lake Bosten, northwestern China. *Aquat. Microb. Ecol.* 69 123–134. 10.3354/ame01627

[B11] Dyksma S. Bischof K. Fuchs B. M. Hoffmann K. Meier D. Meyerdierks A. (2016). Ubiquitous Gammaproteobacteria dominate dark carbon fixation in coastal sediments. *ISME J.* 10 1939–1953. 10.1038/ismej.2015.257 26872043 PMC4872838

[B12] Edlund A. Hårdeman F. Jansson J. K. Sjöling S. (2008). Active bacterial community structure along vertical redox gradients in Baltic Sea sediment. *Environ. Microbiol.* 10 2051–2063. 10.1111/j.1462-2920.2008.01624.x 18452546

[B13] Glöckner F. O. Yilmaz P. Quast C. Gerken J. Beccati A. Ciuprina A. (2017). 25 years of serving the community with ribosomal RNA gene reference databases and tools. *J. Biotechnol.* 261 169–176. 10.1016/j.jbiotec.2017.06.1198 28648396

[B14] Grasshoff K. Ehrhardt M. Kremling K. (eds) (1983). “Methods of seawater analysis,” in *Methods of Seawater Analysis* (Weinheim: Verlag Chemie).

[B15] Haverkamp T. H. Schouten D. Doeleman M. Wollenzien U. Huisman J. Stal L. J. (2009). Colorful microdiversity of *Synechococcus* strains (picocyanobacteria) isolated from the Baltic Sea. *ISME J.* 3 397–408. 10.1038/ismej.2008.118 19052629

[B16] Herlemann D. P. R. Labrenz M. Jürgens K. Bertilsson S. Waniek J. J. Andersson A. F. (2011). Transitions in bacterial communities along the 2000 km salinity gradient of the Baltic Sea. *ISME J.* 5 1571–1579. 10.1038/ismej.2011.41 21472016 PMC3176514

[B17] Herlemann D. P. R. Lundin D. Andersson A. F. Labrenz M. Jürgens K. (2016). Phylogenetic signals of salinity and season in bacterial community composition across the salinity gradient of the Baltic Sea. *Front. Microbiol.* 7:1883. 10.3389/fmicb.2016.01883 27933046 PMC5121245

[B18] Herlemann D. P. R. Lundin D. Labrenz M. Jürgens K. Zheng Z. Aspeborg H. (2013). Metagenomic *de novo* assembly of an aquatic representative of the verrucomicrobial class *Spartobacteria*. *mBio* 4:e00569-12. 10.1128/mBio.00569-12 23716574 PMC3663571

[B19] Herlemann D. P. R. Manecki M. Dittmar T. Jürgens K. (2017). Differential responses of marine, mesohaline and oligohaline bacterial communities to the addition of terrigenous carbon. *Environ. Microbiol.* 19 3098–3117. 10.1111/1462-2920.13784 28474480

[B20] Herlemann D. P. R. Woelk J. Labrenz M. Jürgens K. (2014). Diversity and abundance of “*Pelagibacterales*” (SAR11) in the Baltic Sea salinity gradient. *Syst. Appl. Microbiol.* 37 601–604. 10.1016/j.syapm.2014.09.002 25444644

[B21] Holmes B. (2006). “The genera flavobacterium, Sphingobacterium and weeksella,” in *The Prokaryotes*, eds Dworkin M. Falkow S. Rosenberg E. Schleifer K. Stackebrandt E. (New York, NY: Springer) 539–548.

[B22] Huber J. A. Morrison H. G. Huse S. M. Neal P. R. Sogin M. L. Mark Welch D. B. (2009). Effect of PCR amplicon size on assessments of clone library microbial diversity and community structure. *Environ. Microbiol.* 11 1292–1302. 10.1111/j.1462-2920.2008.01857.x 19220394 PMC2716130

[B23] Hug L. A. Castelle C. J. Wrighton K. C. Thomas B. C. Sharon I. Frischkorn K. R. (2013). Community genomic analyses constrain the distribution of metabolic traits across the *Chloroflexi* phylum and indicate roles in sediment carbon cycling. *Microbiome* 1:22. 10.1186/2049-2618-1-22 24450983 PMC3971608

[B24] Huse S. M. Welch D. M. Morrison H. G. Sogin M. L. (2010). Ironing out the wrinkles in the rare biosphere through improved OTU clustering. *Environ. Microbiol.* 12 1889–1898. 10.1111/j.1462-2920.2010.02193.x 20236171 PMC2909393

[B25] Kan J. Suzuki M. T. Wang K. Evans S. E. Chen F. (2007). High temporal but low spatial heterogeneity of bacterioplankton in the Chesapeake Bay. *Appl. Environ. Microbiol.* 73 6776–6789. 10.1128/AEM.00541-07 17827310 PMC2074944

[B26] Kirchman D. L. Dittel A. I. Malmstrom R. R. Cottrell M. T. (2005). Biogeography of major bacterial groups in the Delaware estuary. *Limnol. Oceanogr.* 50 1697–1706. 10.4319/lo.2005.50.5.1697

[B27] Klindworth A. Pruesse E. Schweer T. Peplies J. Quast C. Horn M. (2012). Evaluation of general 16S ribosomal RNA gene PCR primers for classical and next-generation sequencing-based diversity studies. *Nucleic Acids Res.* 41:e1. 10.1093/nar/gks808 22933715 PMC3592464

[B28] Lenk S. Arnds J. Zerjatke K. Musat N. Amann R. Mussmann M. (2011). Novel groups of *Gammaproteobacteria* catalyse sulfur oxidation and carbon fixation in a coastal, intertidal sediment. *Environ. Microbiol.* 13 758–774. 10.1111/j.1462-2920.2010.02380.x 21134098

[B29] Llobet-Brossa E. Rosselló-Mora R. Amann R. (1998). Microbial community composition of Wadden Sea sediments as revealed by fluorescence in situ hybridization. *Appl. Environ. Microbiol.* 64 2691–2696. 9647850 10.1128/aem.64.7.2691-2696.1998 PMC106446

[B30] Lozupone C. A. Knight R. (2007). Global patterns in bacterial diversity. *Proc. Natl. Acad. Sci. U.S.A.* 104 11436–11440. 10.1073/pnas.0611525104 17592124 PMC2040916

[B31] Ludwig W. Strunk O. Westram R. Richter L. Meier H. Lai T. (2004). ARB: a software environment for sequence data. *Nucleic Acids Res.* 32 1363–1371. 10.1093/nar/gkh293 14985472 PMC390282

[B32] Lv X. Ma B. Yu J. Chang S. X. Xu J. Li Y. (2016). Bacterial community structure and function shift along a successional series of tidal flats in the Yellow River Delta. *Sci. Rep.* 6:36550. 10.1038/srep36550 27824160 PMC5099912

[B33] Marshall I. P. Karst S. M. Nielsen P. H. Jørgensen B. B. (2017). Metagenomes from deep Baltic Sea sediments reveal how past and present environmental conditions determine microbial community composition. *Mar. Genomics* 10.1016/j.margen.2017.08.004 [Epub ahead of print]. 28811148

[B34] McLusky D. S. Elliott M. (2004). *The Estuarine Ecosystem: Ecology, Threats and Management*. London: Oxford University Press on Demand 10.1093/acprof:oso/9780198525080.001.0001

[B35] Naviaux R. K. Good B. Mcpherson J. D. Steffen D. L. Markusic D. Ransom B. (2005). Sand DNA—a genetic library of life at the water’s edge. *Mar. Ecol. Prog. Ser.* 301 9–22. 10.3354/meps301009

[B36] Nealson K. H. (1997). Sediment bacteria: who’s there, what are they doing, and what’s new? *Annu. Rev. Earth Planet Sci.* 25 403–434. 10.1146/annurev.earth.25.1.403 11540735

[B37] Newton R. J. Jones S. E. Eiler A. Mcmahon K. D. Bertilsson S. (2011). A guide to the natural history of freshwater lake bacteria. *Microbiol. Mol. Biol. Rev.* 75 14–49. 10.1128/MMBR.00028-10 21372319 PMC3063352

[B38] Oksanen J. Blanchet F. G. Kindt R. Legendre P. Minchin P. R. O’hara R. (2013). *Package ‘Vegan’. Community Ecology Package, Version 2.0-7*. Available at: http://CRAN.R-project.org/package=vegan

[B39] Pachiadaki M. G. Yakimov M. M. Lacono V. Leadbetter E. Edgcomb V. (2014). Unveiling microbial activities along the halocline of Thetis, a deep-sea hypersaline anoxic basin. *ISME J.* 8 2478–2489. 10.1038/ismej.2014.100 24950109 PMC4260694

[B40] Pavloudi C. Kristoffersen J. B. Oulas A. De Troch M. Arvanitidis C. (2017). Sediment microbial taxonomic and functional diversity in a natural salinity gradient challenge Remane’s “species minimum” concept. *PeerJ* 5:e3687. 10.7717/peerj.3687 29043106 PMC5642246

[B41] Pavloudi C. Oulas A. Vasileiadou K. Sarropoulou E. Kotoulas G. Arvanitidis C. (2016). Salinity is the major factor influencing the sediment bacterial communities in a Mediterranean lagoonal complex (Amvrakikos Gulf, Ionian Sea). *Mar. Genomics* 28 71–81. 10.1016/j.margen.2016.01.005 26831186

[B42] Pruesse E. Peplies J. Glöckner F. O. (2012). SINA: accurate high-throughput multiple sequence alignment of ribosomal RNA genes. *Bioinformatics* 28 1823–1829. 10.1093/bioinformatics/bts252 22556368 PMC3389763

[B43] Pruesse E. Quast C. Knittel K. Fuchs B. M. Ludwig W. Peplies J. (2007). SILVA: a comprehensive online resource for quality checked and aligned ribosomal RNA sequence data compatible with ARB. *Nucleic Acids Res.* 35 7188–7196. 10.1093/nar/gkm864 17947321 PMC2175337

[B44] Quince C. Lanzén A. Curtis T. P. Davenport R. J. Hall N. Head I. M. (2009). Accurate determination of microbial diversity from 454 pyrosequencing data. *Nat. Methods* 6 639–641. 10.1038/nmeth.1361 19668203

[B45] Reichenbach H. (2006). “The order cytophagales,” in *The Prokaryotes* eds Dworkin M. Falkow S. Rosenberg E. Schleifer K. H. Stackebrandt E. (New York, NY: Springer) 549–590.

[B46] Reissmann J. Burchard H. Feistel R. Hagen E. Lass H. U. Mohrholz V. (2009). State-of-the-art review on vertical mixing in the Baltic Sea and consequences for eutrophication. *Prog. Oceanogr.* 82 47–80. 10.1016/j.pocean.2007.10.004

[B47] Remane A. (1934). Die Brackwasserfauna. *Verh. Dtsch. Zool. Ges.* 36 34–74.

[B48] Reyes C. Dellwig O. Dähnke K. Gehre M. Noriega-Ortega B. E. Böttcher M. E. (2016). Bacterial communities potentially involved in iron-cycling in Baltic Sea and North Sea sediments revealed by pyrosequencing. *FEMS Microbiol. Ecol.* 92:fiw054. 10.1093/femsec/fiw054 26960392

[B49] Robertson C. E. Harris J. K. Wagner B. D. Granger D. Browne K. Tatem B. (2013). Explicet: graphical user interface software for metadata-driven management, analysis and visualization of microbiome data. *Bioinformatics* 29 3100–3101. 10.1093/bioinformatics/btt526 24021386 PMC3834795

[B50] Schultz P. Urban N. R. (2008). Effects of bacterial dynamics on organic matter decomposition and nutrient release from sediments: a modeling study. *Ecol. Model.* 210 1–14. 10.1016/j.ecolmodel.2007.06.026

[B51] Seeberg-Elverfeldt J. Schlüter M. Feseker T. Kölling M. (2005). Rhizon sampling of pore waters near the sediment/water interface of aquatic systems. *Limnol. Oceanogr. Methods* 3 361–371. 10.4319/lom.2005.3.361

[B52] Segata N. Izard J. Waldron L. Gevers D. Miropolsky L. Garrett W. S. (2011). Metagenomic biomarker discovery and explanation. *Genome Biol.* 12:R60. 10.1186/gb-2011-12-6-r60 21702898 PMC3218848

[B53] Seidel M. Manecki M. Herlemann D. P. Deutsch B. Schulz-Bull D. Jürgens K. (2017). Composition and transformation of dissolved organic matter in the Baltic Sea. *Front. Earth Sci.* 5:31 10.3389/feart.2017.00031

[B54] Shubenkova O. Likhoshvai A. Kanapatskii T. Pimenov N. (2010). Microbial community of reduced pockmark sediments (Gdansk Deep. Baltic Sea). *Microbiology* 79 799–808. 10.1134/S0026261710060123 21774167

[B55] Sinclair L. Ijaz U. Z. Jensen L. J. Coolen M. J. Gubry-Rangin C. Chroňáková A. (2016). Seqenv: linking sequences to environments through text mining. *PeerJ* 4:e2690. 10.7717/peerj.2690 28028456 PMC5178346

[B56] Stoeck T. Zuendorf A. Breiner H.-W. Behnke A. (2007). A molecular approach to identify active microbes in environmental eukaryote clone libraries. *Microb. Ecol.* 53 328–339. 10.1007/s00248-006-9166-1 17264997

[B57] Telesh I. V. Schubert H. Skarlato S. O. (2011). Revisiting Remane’s concept: evidence for high plankton diversity and a protistan species maximum in the horohalinicum of the Baltic Sea. *Mar. Ecol. Prog. Ser.* 421 1–11. 10.3354/meps08928

[B58] Thomas F. Hehemann J.-H. Rebuffet E. Czjzek M. Michel G. (2011). Environmental and gut bacteroidetes: the food connection. *Front. Microbiol.* 2:93 10.3389/fmicb.2011.00093 PMC3129010 21747801

[B59] Trojan D. Schreiber L. Bjerg J. T. Bøggild A. Yang T. Kjeldsen K. U. (2016). A taxonomic framework for cable bacteria and proposal of the candidate genera Electrothrix and Electronema. *Syst. Appl. Microbiol.* 39 297–306. 10.1016/j.syapm.2016.05.006 27324572 PMC4958695

[B60] Vetterli A. Hyytiäinen K. Ahjos M. Auvinen P. Paulin L. Hietanen S. (2015). Seasonal patterns of bacterial communities in the coastal brackish sediments of the Gulf of Finland, Baltic Sea. *Estuar. Coast. Shelf Sci.* 165 86–96. 10.1016/j.ecss.2015.07.049

[B61] Walsh D. A. Lafontaine J. Grossart H.-P. (2013). “On the eco-evolutionary relationships of fresh and salt water bacteria and the role of gene transfer in their adaptation,” in *Lateral Gene Transfer in Evolution* ed. Gophna U. (New York, NY: Springer) 55–77.

[B62] Wang Y. Sheng H.-F. He Y. Wu J.-Y. Jiang Y.-X. Tam N. F.-Y. (2012). Comparison of the levels of bacterial diversity in freshwater, intertidal wetland, and marine sediments by using millions of illumina tags. *Appl. Environ. Microbiol.* 78 8264–8271. 10.1128/AEM.01821-12 23001654 PMC3497375

[B63] Zettler M. L. Karlsson A. Kontula T. Gruszka P. Laine A. O. Herkül K. (2014). Biodiversity gradient in the Baltic Sea: a comprehensive inventory of macrozoobenthos data. *Helgol. Mar. Res.* 68 49–57. 10.1007/s10152-013-0368-x

[B64] Zhang J. Yang Y. Zhao L. Li Y. Xie S. Liu Y. (2015). Distribution of sediment bacterial and archaeal communities in plateau freshwater lakes. *Appl. Microbiol. Biotechnol.* 99 3291–3302. 10.1007/s00253-014-6262-x 25432677

[B65] Zheng B. Wang L. Liu L. (2014). Bacterial community structure and its regulating factors in the intertidal sediment along the Liaodong Bay of Bohai Sea. China. *Microbiol. Res.* 169 585–592. 10.1016/j.micres.2013.09.019 24231160

[B66] Zhou J. Wu L. Deng Y. Zhi X. Jiang Y.-H. Tu Q. (2011). Reproducibility and quantitation of amplicon sequencing-based detection. *ISME J.* 5 1303–1313. 10.1038/ismej.2011.11 21346791 PMC3146266

[B67] Zinger L. Amaral-Zettler L. A. Fuhrman J. A. Horner-Devine M. C. Huse S. M. Welch D. B. M. (2011). Global patterns of bacterial beta-diversity in seafloor and seawater ecosystems. *PLoS One* 6:e24570. 10.1371/journal.pone.0024570 21931760 PMC3169623

