# Supplementary material for: Benthic Bacterial Community Composition in the Oligohaline-Marine Transition of Surface Sediments in the Baltic Sea Based on rRNA Analysis
Source: Front Microbiol. 2018 Feb 19;9:236. doi: 10.3389/fmicb.2018.00236 (PMC5827536; doi:10.3389/fmicb.2018.00236)
Supplement: Supplementary file 1 [file Table_1.pdf]

Table S1. Sediment properties including grain size (Q_{50}), total nitrogen (TN), total sulfur (TSul), and total organic carbon (TOC) concentrations in the samples. The abbreviation indicates the station: A (salinity 35); B (salinity 21); C (salinity 8); D (salinity 7); E (salinity 4) and the sediment horizon (A1-C see Fig. 5).

	**Q_{50}**	**TN [%]**	**TSul [%]**	**TOC [%]**	**TOC/N**
A_I_A1	5.57	0.31	0.32	2.4	7.9
A_I_A2	4.64	0.29	0.33	2.53	8.7
A_I_B1	5.03	0.29	0.33	2.45	8.6
A_I_B2	5.79	0.29	0.30	2.47	8.6
A_I_C	4.44	0.29	0.34	2.53	8.8
A_II_A1	6.23	0.3	0.27	2.5	8.3
A_II_A2	4.23	0.3	0.26	2.47	8.3
A_II_B1	5.11	0.31	0.31	2.63	8.5
A_II_C	5.36	0.29	0.27	2.53	8.7
A_III_A1	4.95	0.29	0.32	2.58	8.8
A_III_A2	4.47	0.29	0.30	2.42	8.2
A_III_B1	4.67	0.28	0.31	2.46	8.7
A_III_B2	4.91	0.3	0.29	2.57	8.7
A_III_C	5.4	0.3	0.27	2.62	8.8
B_I_A1	8.97	0.49	0.66	3.97	8.1
B_I_A2	8.89	0.49	0.73	3.88	7.9
B_I_B1	11.26	0.42	0.82	3.41	8.1
B_I_B2	15.36	0.49	0.90	4.02	8.2
B_I_C	9.27	0.53	1.09	4.29	8
B_II_A1	13.6	0.52	0.79	4.12	8
B_II_A2	10.42	0.52	0.71	4.17	8
B_II_B1	9.22	0.48	0.62	3.78	8
B_II_B2	10.83	0.54	0.75	4.31	8
B_II_C	9.88	0.5	0.73	4.07	8.2
B_III_A1	8.5	0.49	0.72	3.99	8.1
B_III_A2	9.71	0.53	0.83	4.16	7.9
B_III_B1	12.49	0.52	0.99	4.2	8.1
B_III_B2	8.6	0.44	0.87	3.69	8.4
B_III_C	13.6	0.5	1.03	4.1	8.3

Sample	Q_{50}	TN [%]	TSul [%]	TOC [%]	TOC/N
C_I_A1	13.1	0.87	0.38	6.48	7.5
C_I_A2	13.22	0.31	0.25	2.47	7.9
C_I_B1	15.03	0.69	0.38	5.06	7.4
C_I_B2	10.25	0.53	0.47	3.81	7.2
C_I_C	9.79	0.44	0.37	3.3	7.5
C_II_A1	12.4	0.19	0.13	1.36	7.3
C_II_A2	12.5	0.15	0.07	1.08	7
C_II_B1	7.44	0.14	0.07	1.02	7.5
C_II_B2	7.64	0.09	0.08	0.69	7.7
C_II_C	7.83	0.09	0.05	0.67	7.4
C_III_A1	15.82	0.07	0.02	0.45	6.8
C_III_A2	7.74	0.05	0.02	0.41	7.8
C_III_B1	6.98	0.07	0.03	0.48	6.6
C_III_B2	6.47	0.05	0.02	0.36	7.1
C_III_C	36.43	0.05	0.02	0.35	7.2
D_I_A2	2.78	0.05	0.03	0.43	8.6
D_I_B1	2.69	0.05	0.01	0.47	9.4
D_II_A1	203.84	0.06	0.03	0.49	8.2
D_II_A2	186.42	0.06	0.02	0.48	8
D_II_B1	172.35	0.04	0.02	0.34	8.4
D_II_B2	205.8	0.02	0.00	0.2	10.2
D_III_A1	377.36	0.04	0.03	0.41	10.2
D_III_A2	3.26	0.02	0.02	0.22	10.9
D_III_B2	2.93	0.05	0.02	0.52	10.4
E_I_A1	10.04	0.47	0.14	4.87	10.4
E_I_A2	10.52	0.45	0.13	4.46	10
E_I_B1	10.24	0.43	0.13	4.03	9.5
E_I_B2	8.18	0.4	0.14	3.83	9.5
E_II_A1	11.8	0.46	0.14	4.68	10.2
E_II_A2	9.24	0.43	0.13	4.03	9.4
E_II_B1	12.04	0.41	0.18	3.9	9.6
E_II_B2	10.67	0.39	0.23	3.8	9.8
E_III_A1	9.77	0.47	0.15	4.93	10.5
E_III_A2	7.88	0.46	0.13	4.73	10.2
E_III_B1	10.85	0.44	0.12	4.38	9.9
E_III_B2	9.17	0.42	0.14	4.07	9.8
E_III_C	9.16	0.4	0.16	3.89	9.6

Table S2. Porewater chemistry in the different sediment samples, including H_2S, Fe_{diss}, Mn, PO_4, SO_4, and $Si(OH)_3$. Abbreviation refer to the stations: A (salinity 35); B (salinity 21); C (salinity 8); D (salinity 7); E (salinity 4).

Station	Bottom Salinity	Core depth [cm]	H_2S [µM]	Fe [µM]	Mn [µM]	PO_4 [µM]	SO_4^{2-} [mM]	$Si(OH)_3$ [µM]	NH_4^+ [µM]	NO_2^- [µM]	NO_3^- [µM]
A	35	-5	0	0.6	0.0	1.1	28.4	12	2	0	9.4
A	35	0.5	0	0.4	0.0	1.6	29.6	19	2.7	0	9.9
A	35	1.5	0	0.4	2.5	2.5	29.3	83	3.1	0.1	7.6
A	35	2.5	0	0.1	19.1	2.0	30.1	60	3.5	0	6.2
A	35	3.5	0	0.1	34.2	1.9	29.8	118	12.7	0.1	4.2
A	35	4.5	0	2.0	81.4	2.2	29.5	137	16.5	0.2	3.1
A	35	5.5	0	0.3	118.4	3.6	29.6	167	25.7	0.1	2.1
A	35	6.5	0	17.0	135.2	5.2	29.8	135	26.4	0.1	1.3
A	35	7.5	0	29.4	103.4	6.5	30.0	117	33.9	0.1	0.7
A	35	8.5	0	80.1	95.3	15.9	29.7	135	38.6	0.1	0.8
A	35	9.5	0	97.1	87.8	20.2	29.4	133	47.4	0.1	1.1
A	35	10.5	0	90.6	83.8	24.8	30.0	144	55.4	0.1	1.2
A	35	11.5	0	86.7	81.1	22.5	29.2	139	58.9	0.1	0.7
A	35	12.5	0	76.6	86.0	23.8	30.1	146	70	0.1	0.5
A	35	13.5	0	83.4	93.9	27.8	30.1	158	73.1	0	0.7
A	35	15.5	0	94.2	112.3	37.9	29.9	177	95.6	0.1	0.5
A	35	17.5	0	123.3	124.9	49.4	29.4	178	130.1	0.1	0.7
A	35	19.5	0	176.9	135.8	68.9	29.3	192	162.2	0.2	1.1
A	35	21.5	0	171.9	145.6	74.1	29.6	210	184.4	0.1	0.8
A	35	23.5	0	145.4	150.3	73.8	28.7	239	211.6	0.2	1.1
A	35	25.5	0	119.3	156.3	76.7	28.8	271	239	0.3	1
A	35	27.5	0	101.9	156.5	76.2	28.1	312	261.6	0.3	1.2
A	35	29.5	0	74.5	153.8	72.5	27.1	358	286.5	0.3	1
B	21	-5	0	0.3	0.4	1.7	16.1	37	29	0.4	2.6
B	21	0.5	0	52.8	11.9	6.8	14.0	185	40.2	0.4	1
B	21	1.5	0	31.9	14.9	28.0	16.1	116	66.8	0.3	1.9
B	21	2.5	0	31.0	19.7	47.5	15.4	447	160.4	0.3	0.9
B	21	3.5	0	25.4	21.0	56.3	16.4	290	102.5	0.6	0.6
B	21	4.5	8	17.9	21.6	64.9	15.2	537	204.6	0.2	0.7
B	21	5.5	42	11.2	22.3	83.5	16.2	361	133.6	0.1	0.9
B	21	6.5	47	9.3	23.1	86.5	15.9	578	240.8	0.2	0.5
B	21	7.5	119	4.3	23.2	74.6	16.5	478	214	0.1	1.9
B	21	8.5	244	1.7	23.9	94.4	15.6	655	301.4	0.1	0.6
B	21	9.5	271	1.4	24.1	93.6	16.0	631	294	0.4	0.7
B	21	11.5	315	0.7	25.4	105.1	15.2	686	332.1	0.2	0.5

Station	Bottom Salinity	Core depth [cm]	H_2S [µM]	Fe [µM]	Mn [µM]	PO_4 [µM]	SO_4^{2-} [mM]	$Si(OH)_3$ [µM]	NH_4^+ [µM]	NO_2^- [µM]	NO_3^- [µM]
B	21	19.5	423	0.7	27.4	119.9	13.7	731	450.8	0.2	0.7
B	21	23.5	557	0.7	34.9	145.3	13.1	729	527.9	0.3	0.7
B	21	27.5	1080	0.4	35.5	139.3	13.4	750	559.1	0.1	0.2
D	2	0.5	0	5.1	20.7	3.0	5.9	66	0.1	0	2.6
D	2	1.5	0	2.0	17.6	4.3	6.2	69	1.3	0	2.7
D	2	2.5	0	0.2	13.9	4.3	6.0	68	2.4	0	2.8
D	2	3.5	0	0.7	12.3	1.7	6.0	87	4.9	0	3.1
D	2	4.5	0	3.6	11.6	2.6	6.1	97	6.8	0	3.2
D	2	5.5	0	5.2	11.4	2.3	6.2	102	7.2	0	5
D	2	6.5	0	7.4	9.6	2.2	6.1	114	7	0	3.1
D	2	8.5	0	12.3	8.7	3.1	6.2	125	6	0	1.1
D	2	10.5	0	12.1	8.1	3.0	5.5	134	NA	NA	NA
D	2	12.5	0	15.6	7.7	3.0	5.6	146	NA	NA	NA
D	2	14.5	0	16.4	7.6	3.1	5.8	156	NA	NA	NA
D	2	16.5	0	20.2	7.0	3.9	5.9	159	NA	NA	NA
D	2	18.5	0	21.9	6.9	3.9	5.9	171	NA	NA	NA
D	2	20.5	0	21.6	6.7	3.5	5.8	163	NA	NA	NA
D	2	22.5	0	21.0	6.6	3.0	5.8	168	NA	NA	NA

Table S3.Tukey test of the accumulated number of bacterial genera at the different stations. Tukey's Q values are below the diagonal and *p* above the diagonal. Significant comparison are in italics. A (salinity 35); B (salinity 21); C (salinity 8); D (salinity 7); E (salinity 4) (1-15 see Fig. 5)

	A	B	C	D	E
A		0.8567	*0.005036*	0.6353	*0.02267*
B	1.407		*0.0002972*	0.9947	0.2247
C	5.149	6.556		*0.0001684*	*0.0001325*
D	1.968	0.5614	7.118		0.4273
E	4.406	2.999	9.556	2.438	

Table S4.Result of the linear discriminant analysis effect size (LEfSe) analysis. Listed are all bacterial genera, their score in the least discriminate analysis (LDA) and their relative rRNA abundance in the dataset. The highest abundances are in bold. The stations were abbreviated as A (salinity 35); B (salinity 21); C (salinity 8); D (salinity 7); E (salinity 4). See Figure 1 for details.

	LDA-score		A	B	C	D	E	Average abundance
Bacteroidetes/VC2.1 Bac22/unclassified/unclassified/unclassified	3.84	A	**0.70**	0.36	0.24	0.10	0.03	0.29
Fibrobacteres/Fibrobacteria/Fibrobacterales/09D2Z46/unclassified	4.15	A	**1.42**	0.68	0.45	0.24	0.10	**0.58**
Proteobacteria/Gammaproteobacteria/Chromatiales/Chromatiaceae/uncultured	4.11	A	**1.29**	0.86	0.01	0.00	0.00	0.43
Proteobacteria/Gammaproteobacteria/Order Incertae Sedis/Family Incertae Sedis/Thiohalomonas	4.17	A	**1.47**	0.14	0.01	0.00	0.00	0.32
Proteobacteria/Gammaproteobacteria/Thiotrichales/Piscirickettsiaceae/endosymbionts	3.57	A	**0.37**	0.26	0.04	0.07	0.00	0.15
Firmicutes/Clostridia/Halanaerobiales/64K2/unclassified	3.89	B	0.43	**0.77**	0.24	0.16	0.10	0.34
Fusobacteria/Fusobacteriia/Fusobacteriales/Fusobacteriaceae/Psychrilyobacter	3.30	B	0.02	**0.20**	0.01	0.01	0.00	0.05
Lentisphaerae/Lentisphaeria/R76-B128/unclassified/unclassified	3.87	B	0.39	**0.73**	0.26	0.34	0.14	0.37
Proteobacteria/Deltaproteobacteria/Desulfobacterales/Desulfobacteraceae/Sva0081 sediment group	4.68	B	2.86	**4.73**	1.86	0.89	0.33	**2.14**
Proteobacteria/Deltaproteobacteria/Desulfobacterales/Desulfobulbaceae/Ca. Electrothrix	3.54	B	0.19	**0.34**	0.08	0.00	0.02	0.13
Proteobacteria/Deltaproteobacteria/Syntrophobacterales/Syntrophobacteraceae/uncultured	3.87	B	0.14	**0.73**	0.00	0.02	0.08	0.19
Proteobacteria/Epsilonproteobacteria/Campylobacterales/Helicobacteraceae/Sulfurimonas	4.03	B	0.24	**1.08**	0.10	0.01	0.01	0.29
Proteobacteria/Gammaproteobacteria/BD7-8 marine group/unclassified/unclassified	4.32	B	1.41	**2.10**	0.82	0.47	0.26	**1.01**
Proteobacteria/Gammaproteobacteria/Chromatiales/Ectothiorhodospiraceae/Acidiferrobacter	5.10	B	4.62	**12.52**	1.36	4.38	0.08	**4.59**
Proteobacteria/Gammaproteobacteria/Chromatiales/Ectothiorhodospiraceae/Thioalkalispira	3.95	B	0.46	**0.90**	0.11	0.13	0.03	0.33
Proteobacteria/Gammaproteobacteria/Chromatiales/Granulosicoccaceae/Granulosicoccus	4.07	B	0.16	**1.19**	0.03	0.01	0.00	0.28
Proteobacteria/Gammaproteobacteria/Order Incertae Sedis/Family Incertae Sedis/Thiohalophilus	4.20	B	1.15	**1.58**	0.03	0.01	0.01	**0.56**
Proteobacteria/Gammaproteobacteria/Thiotrichales/Thiotrichaceae/uncultured	4.85	B	5.02	**7.08**	0.60	0.09	0.11	**2.58**
Proteobacteria/unclassified/unclassified/unclassified/unclassified	3.28	B	0.04	**0.19**	0.02	0.01	0.00	0.05
Bacteroidetes/Sphingobacteriia/Sphingobacteriales/Saprospiraceae/Portibacter	3.18	C	0.03	0.00	**0.15**	0.15	0.05	0.08
Cyanobacteria/Cyanobacteria/SubsectionIV/FamilyI/unclassified	3.85	C	0.05	0.26	**0.71**	0.23	0.00	0.25
Cyanobacteria/Cyanobacteria/SubsectionI/FamilyI/unclassified	4.49	D	0.14	0.07	0.33	**3.07**	0.00	**0.72**
Cyanobacteria/Cyanobacteria/SubsectionIV/FamilyI/Anabaena	4.47	D	0.09	0.28	0.88	**2.97**	0.00	**0.84**
Proteobacteria/Gammaproteobacteria/Alteromonadales/Psychromonadaceae/Psychromonas	3.68	D	0.14	0.09	0.34	**0.48**	0.06	0.22
Bacteroidetes/vadinHA17/unclassified/unclassified/unclassified	4.47	E	0.11	0.06	0.73	0.08	**2.95**	**0.79**
Chlorobi/Chlorobia/Chlorobiales/OPB56/unclassified	3.97	E	0.08	0.05	0.25	0.63	**0.92**	0.39
Chloroflexi/S085/unclassified/unclassified/unclassified	2.95	E	0.00	0.01	0.04	0.00	**0.09**	0.03
Proteobacteria/Alphaproteobacteria/Rhizobiales/Family Incertae Sedis/Bauldia	3.86	E	0.00	0.01	0.13	0.30	**0.72**	0.23
Proteobacteria/Betaproteobacteria/TRA3-20/unclassified/unclassified	3.61	E	0.15	0.00	0.07	0.11	**0.40**	0.15
Proteobacteria/Deltaproteobacteria/GR-WP33-30/unclassified/unclassified	3.96	E	0.14	0.08	0.51	0.88	**0.90**	**0.50**

Table S5. EnvO terms of the abundant operational taxonomic units (OTU) based on 16 rRNA analysis. The most abundant rRNA sequence from an OTU was used in the *seqenv* analysis. The number of EnvO terms obtained based on the analysis is shown.

	sediment	sea	marine sediment	soil	biofilm	ocean	mud volcano
09D2Z46 OTU	**28**	**10**	0	0	0	**2**	0
Acidiferrobacter OTU	**30**	**12**	**1**	0	0	0	0
Anaerolineaceae OTU	**4**	**4**	**1**	0	0	**1**	**3**
BD2-2 OTU	**12**	0	**1**	**1**	0	0	**6**
BD7-8 OTU	**6**	0	**28**	0	0	0	0
Candidatus Alysiosphaera OTU	**4**	**4**	0	0	**9**	0	0
Desulfobacteraceae OTU	0	0	0	0	**2**	0	0
Ca. Electrothrix OTU	**14**	0	0	0	0	0	0
GR-WP33-58 OTU	**9**	**1**	0	0	**1**	0	0
JG30-KF OTU	**2**	0	0	0	0	0	0
JTB255 OTU	**15**	**24**	0	0	0	0	0
KD4-96 OTU	**4**	**1**	0	**14**	0	0	0
Magnetococcus OTU	0	**1**	0	0	0	0	0
Marinicella OTU	**30**	**1**	0	0	**1**	0	0
MSBL5 OTU	**20**	**1**	**1**	0	0	**1**	0
Nkb17 OTU	**24**	**24**	0	0	0	**1**	0
Candidatus Parabegiatoa OTU	**1**	0	**2**	0	0	0	0
Pelagibius OTU	**6**	**6**	0	0	0	0	0
Rubricoccus OTU	**1**	0	**1**	0	0	**6**	0
SAR202 OTU	0	0	0	**6**	0	0	0
SEEP-SRB1 OTU	**16**	**4**	**4**	0	0	0	0
Subgroup 22 OTU	**36**	**4**	**4**	0	0	0	0
Sva0081 OTU	**21**	0	**4**	0	0	0	0
Thiohalophilus OTU	**9**	0	**1**	0	0	0	0
Thiotrichaeceae OTU	**1**	**12**	0	0	0	0	**1**
vadinHA17 OTU	**12**	0	0	0	0	0	0
WCHB1-41 OTU	**12**	**1**	**1**	0	0	0	0
**Total**	**317**	**110**	**49**	**21**	**13**	**11**	**10**

	beach	ENVO:00000447	marine habitat	aquatic habitat	ENVO:00002030	intertidal sediment	petroleum
09D2Z46 OTU	0	**1**	**1**	0	0	0	0
Acidiferrobacter OTU	0	**1**	**1**	0	0	0	0
Anaerolineaceae OTU	**1**	0	0	0	0	0	0
BD2-2 OTU	0	**1**	**1**	0	0	0	0
BD7-8 OTU	**6**	0	0	0	0	0	0
Candidatus Alysiosphaera OTU	0	0	0	0	0	0	0
Desulfobacteraceae OTU	0	0	0	**1**	**1**	0	0
Ca. Electrothrix OTU	0	0	0	0	0	0	0
GR-WP33-58 OTU	0	**1**	**1**	0	0	0	0
JG30-KF OTU	0	0	0	0	0	0	0
JTB255 OTU	0	0	0	0	0	**1**	0
KD4-96 OTU	0	0	0	0	0	0	0
Magnetococcus OTU	0	0	0	0	0	**1**	0
Marinicella OTU	0	0	0	0	0	0	0
MSBL5 OTU	0	0	0	0	0	0	0
Nkb17 OTU	0	0	0	0	0	0	0
Candidatus Parabegiatoa OTU	0	**1**	**1**	0	0	0	0
Pelagibius OTU	0	0	0	0	0	0	0
Rubricoccus OTU	0	0	0	0	0	0	0
SAR202 OTU	0	0	0	0	0	0	0
SEEP-SRB1 OTU	0	0	0	0	0	0	**1**
Subgroup 22 OTU	**1**	**1**	**1**	0	0	0	0
Sva0081 OTU	**1**	**1**	**1**	0	0	0	**4**
Thiohalophilus OTU	0	**1**	**1**	0	0	0	0
Thiotrichaeceae OTU	0	0	0	0	0	**5**	0
vadinHA17 OTU	0	0	0	**6**	**6**	0	**2**
WCHB1-41 OTU	0	0	0	0	0	0	0
**Total**	**9**	**8**	**8**	**7**	**7**	**7**	**7**

	hydrothermal vent	wetland	ground water	lake sediment	cave	coast	mediterranean
09D2Z46 OTU	0	0	0	0	0	0	0
Acidiferrobacter OTU	**1**	0	0	0	0	0	0
Anaerolineaceae OTU	0	0	0	**3**	0	0	**3**
BD2-2 OTU	0	0	0	0	0	0	0
BD7-8 OTU	0	0	0	0	0	**1**	0
Candidatus Alysiosphaera OTU	**1**	0	0	0	0	0	0
Desulfobacteraceae OTU	0	0	**2**	0	**2**	0	0
Ca. Electrothrix OTU	0	0	0	0	0	0	0
GR-WP33-58 OTU	0	0	0	0	0	0	0
JG30-KF OTU	0	0	0	0	0	0	0
JTB255 OTU	**1**	0	0	0	0	0	0
KD4-96 OTU	0	0	**1**	**1**	0	0	0
Magnetococcus OTU	0	0	0	0	0	0	0
Marinicella OTU	0	0	0	0	**1**	0	0
MSBL5 OTU	0	0	0	0	0	0	0
Nkb17 OTU	0	0	0	0	0	0	0
Candidatus Parabegiatoa OTU	0	0	0	0	0	0	0
Pelagibius OTU	0	0	**1**	0	0	0	0
Rubricoccus OTU	0	0	0	0	0	**1**	0
SAR202 OTU	0	0	0	0	0	0	0
SEEP-SRB1 OTU	**1**	0	0	0	0	0	0
Subgroup 22 OTU	0	0	0	0	0	0	0
Sva0081 OTU	0	0	0	0	0	0	0
Thiohalophilus OTU	**1**	0	0	0	0	0	0
Thiotrichaeceae OTU	0	0	0	0	0	**1**	0
vadinHA17 OTU	0	**6**	0	0	0	0	0
WCHB1-41 OTU	**1**	0	0	0	0	0	0
**Total**	**6**	**6**	**4**	**4**	**3**	**3**	**3**

	oil seep	activated sludge	bay	cold seep	ENVO:01000047	fresh water
09D2Z46 OTU	0	0	0	**1**	0	0
Acidiferrobacter OTU	0	0	0	0	0	0
Anaerolineaceae OTU	0	0	0	**1**	0	0
BD2-2 OTU	0	0	0	0	**1**	0
BD7-8 OTU	0	0	0	0	0	0
Candidatus Alysiosphaera OTU	0	**1**	0	0	0	0
Desulfobacteraceae OTU	0	0	0	0	0	0
Ca. Electrothrix OTU	0	0	0	0	0	0
GR-WP33-58 OTU	0	0	0	0	0	0
JG30-KF OTU	0	0	0	0	0	0
JTB255 OTU	0	0	0	0	0	0
KD4-96 OTU	0	0	0	0	0	0
Magnetococcus OTU	0	0	0	0	0	**2**
Marinicella OTU	0	0	0	0	0	0
MSBL5 OTU	0	0	0	0	0	0
Nkb17 OTU	0	0	0	0	0	0
Candidatus Parabegiatoa OTU	0	0	0	0	0	0
Pelagibius OTU	0	**1**	0	0	0	0
Rubricoccus OTU	0	0	0	0	0	0
SAR202 OTU	0	0	**1**	0	0	0
SEEP-SRB1 OTU	0	0	0	0	0	0
Subgroup 22 OTU	0	0	0	0	0	0
Sva0081 OTU	0	0	0	0	0	0
Thiohalophilus OTU	0	0	0	0	0	0
Thiotrichaeceae OTU	**1**	0	**1**	0	**1**	0
vadinHA17 OTU	0	0	0	0	0	0
WCHB1-41 OTU	**2**	0	0	0	0	0
**Total**	**3**	**2**	**2**	**2**	**2**	**2**
