# Supplementary material for: Benthic Bacterial Community Composition in the Oligohaline-Marine Transition of Surface Sediments in the Baltic Sea Based on rRNA Analysis
Source: Front Microbiol. 2018 Feb 19;9:236. doi: 10.3389/fmicb.2018.00236 (PMC5827536; doi:10.3389/fmicb.2018.00236)
Supplement: Supplementary file 2 [file Image_1.PDF]

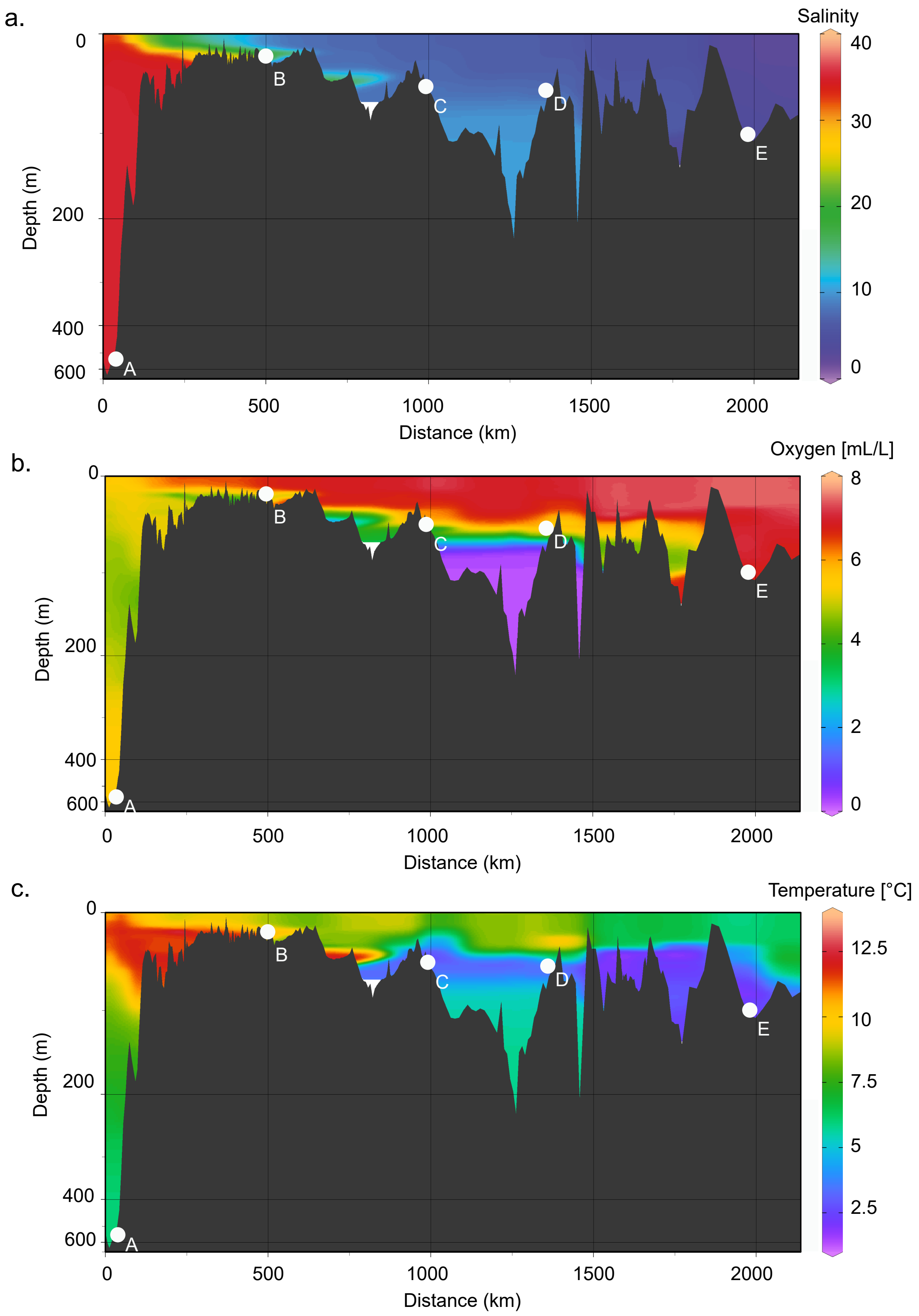

Figure S1. Contour depth plot of (a) salinity, (b) oxygen, and (c) temperature for the sampled transect at stations A, B, C, D, and E (see Fig. 1). Station A has the internal name Geo2; station B has the internal name S4; station C has the intenal name Mo15, station D has the internal name S8, station E has the internal name S11.

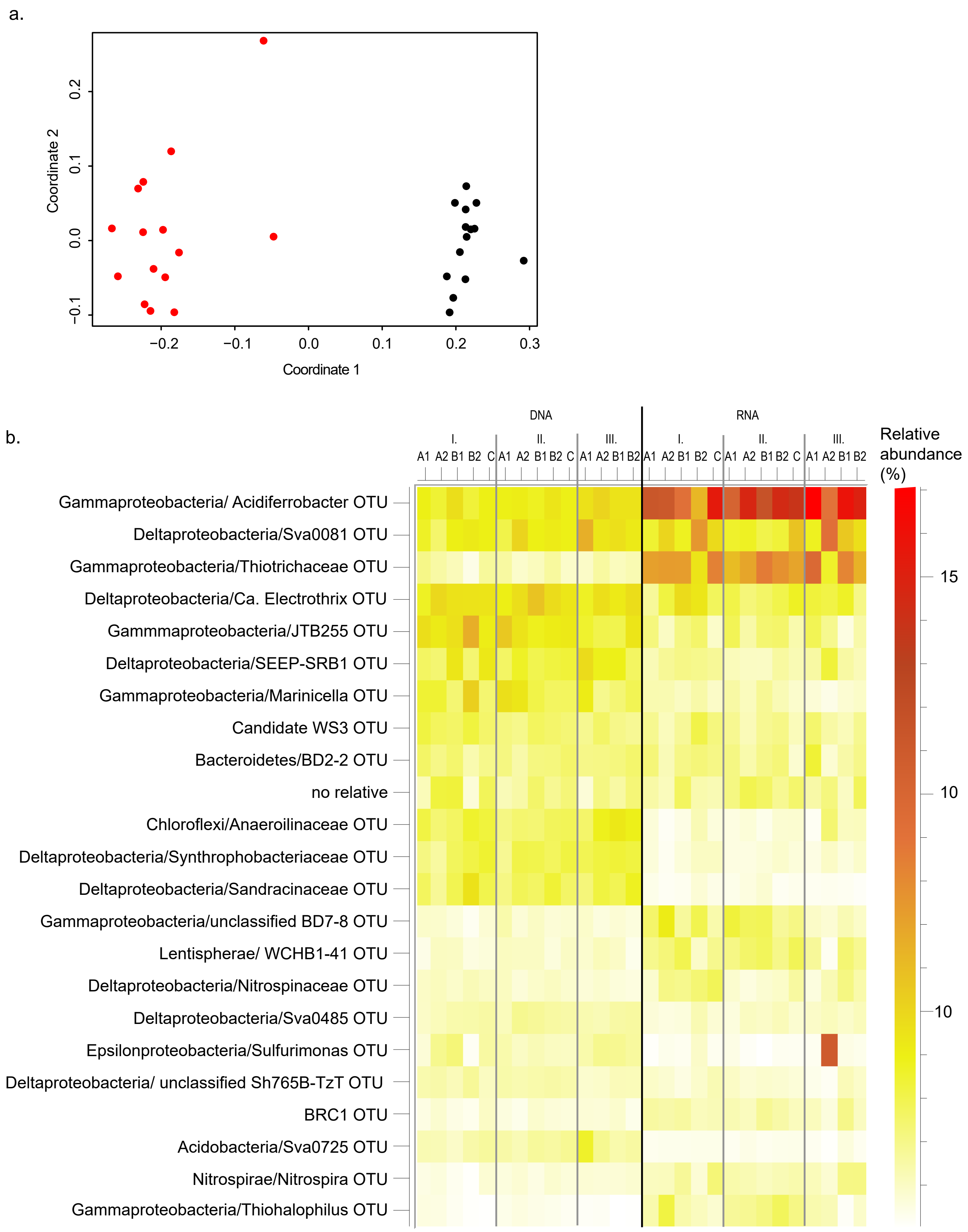

Figure S2. Comparison of the DNA (black)- and RNA- (red) based bacterial community composition at station B. (a) Principle coordinate analysis (PCoA) plot based on Bray-Curtis similarity; (b) heatmap of the abundant OTUs in the sample.

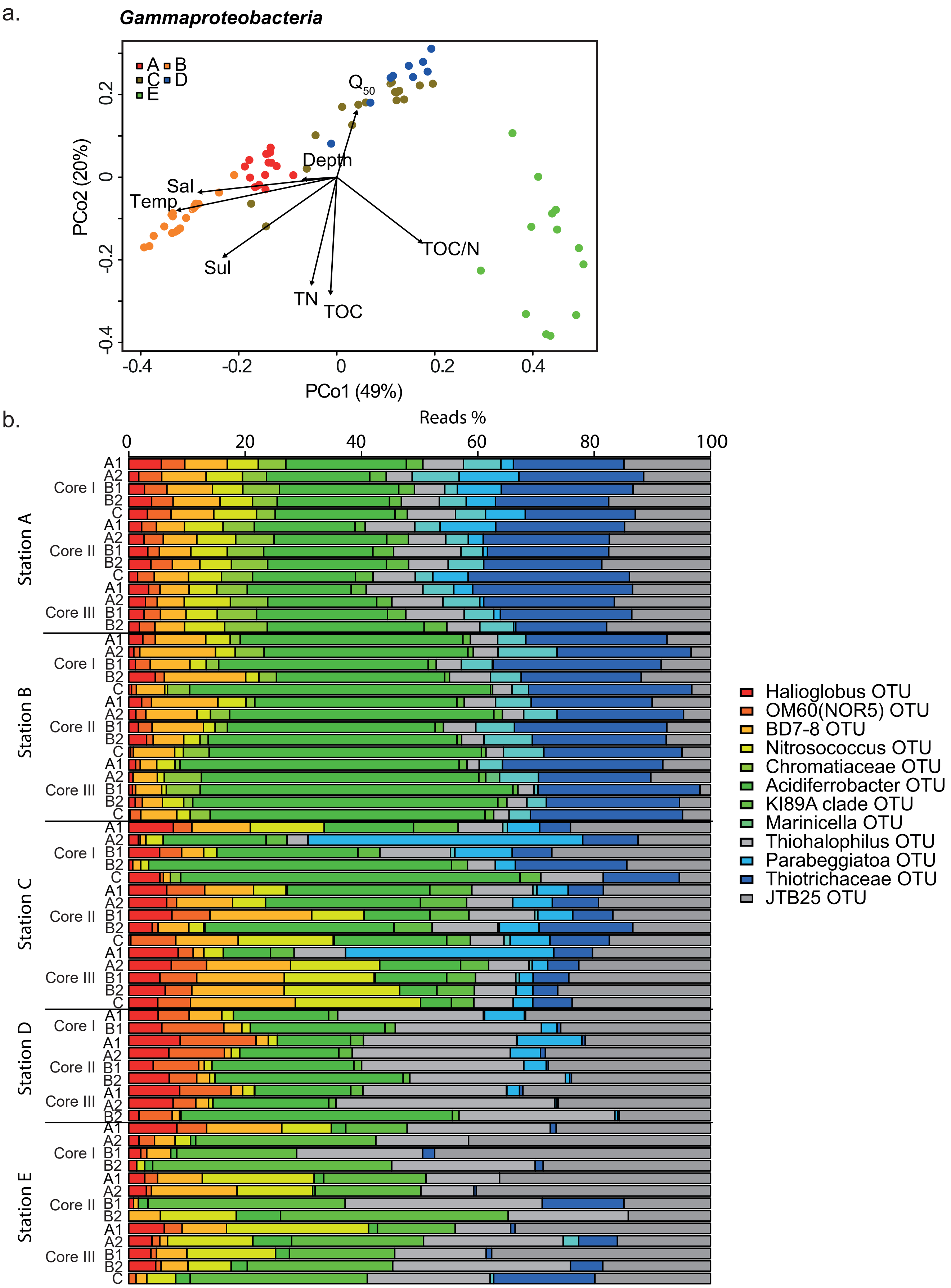

Figure S3. Analysis of the 16S rRNA assigned to the *Gammaproteobacteria*. (a) PCoA plot based on the Bray-Curtis dissimilarity index. (b) Abundance patterns for all OTUs assigned to Gammaproteobacteria are shown as stacked bar graphs. For clarity, the relative abundance cut-off level was 1.8% for each individual OTU assigned to the *Gammaproteobacteria*.

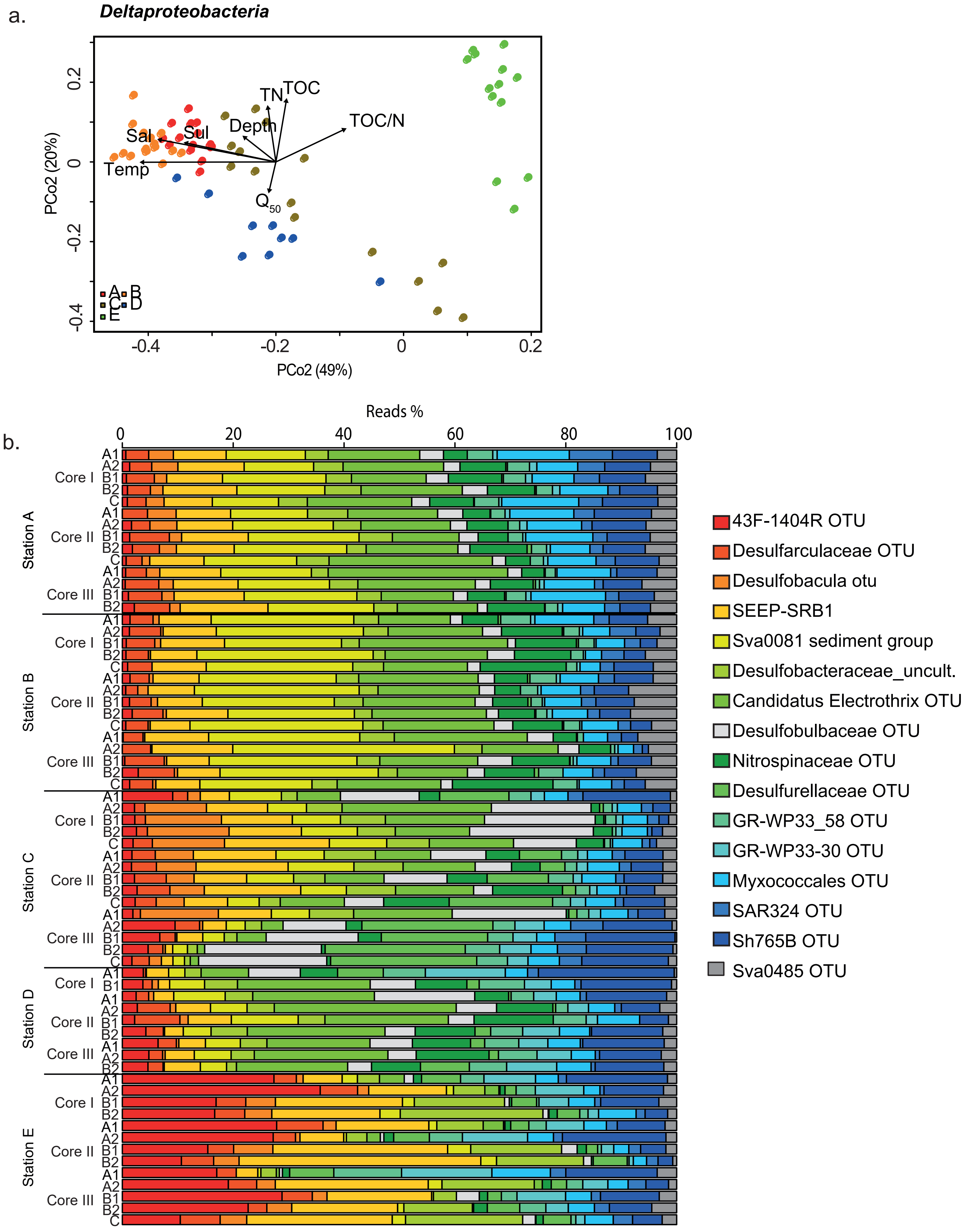

Figure S4. Analysis of the 16S rRNA assigned to the *Deltaproteobacteria*. (a) PCoA plot based on the Bray-Curtis dissimilarity index. (b) The abundance patterns of all OTUs assigned to *Deltaproteobacteria* are shown as stacked bar graphs. For clarity, the relative abundance cut-off level was 1.8% for each individual OTU assigned to the *Deltaproteobacteria*.

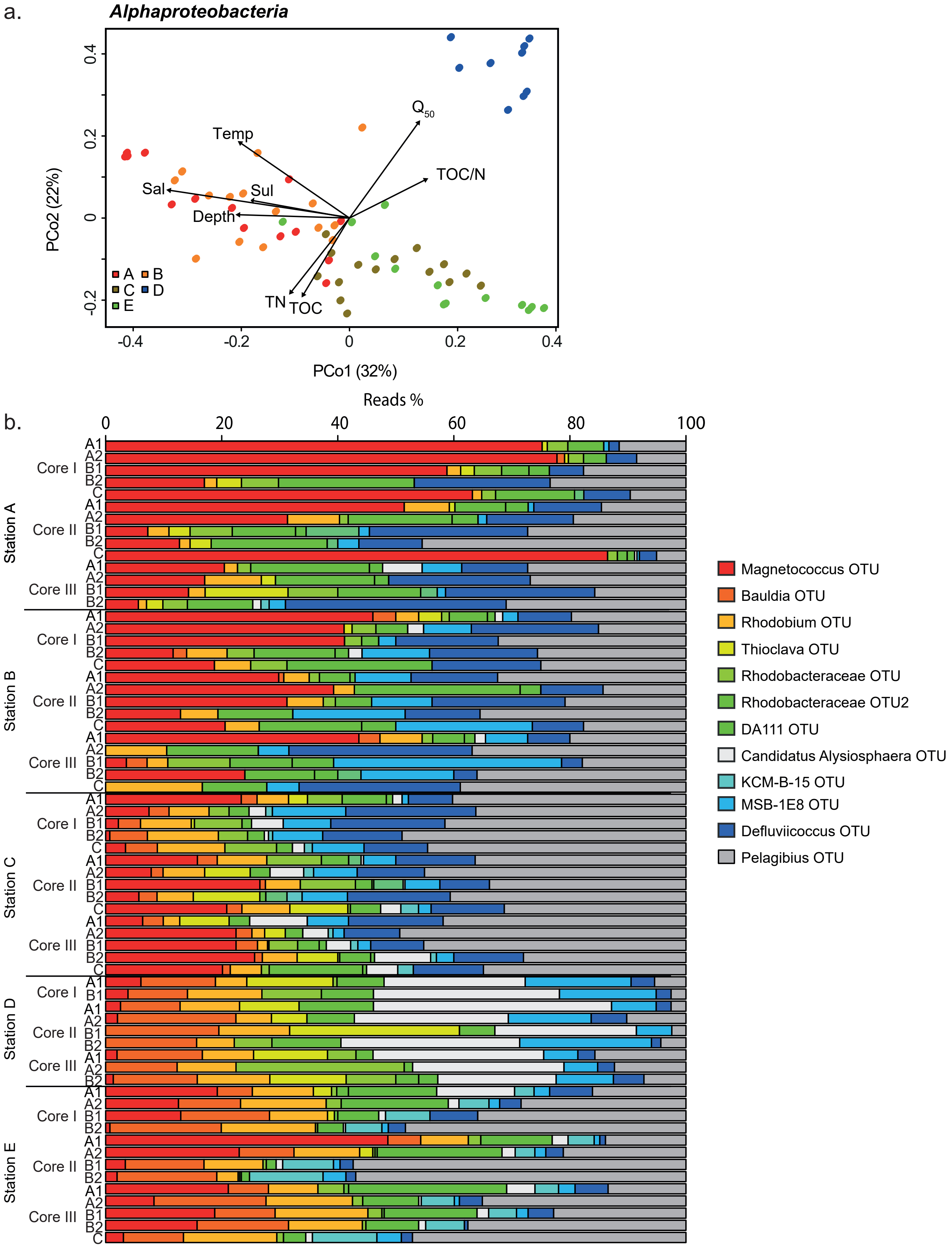

Figure S5. Analysis of the 16S rRNA assigned to the *Alphaproteobacteria*. (a) PCoA plot based on the Bray-Curtis dissimilarity index. (b) The abundance patterns of all OTUs assigned to the *Alphaproteobacteria* are shown as stacked bar graphs. For clarity, the relative abundance cut-off level was 1.8% for each individual OTU assigned to the *Alphaproteobacteria*.

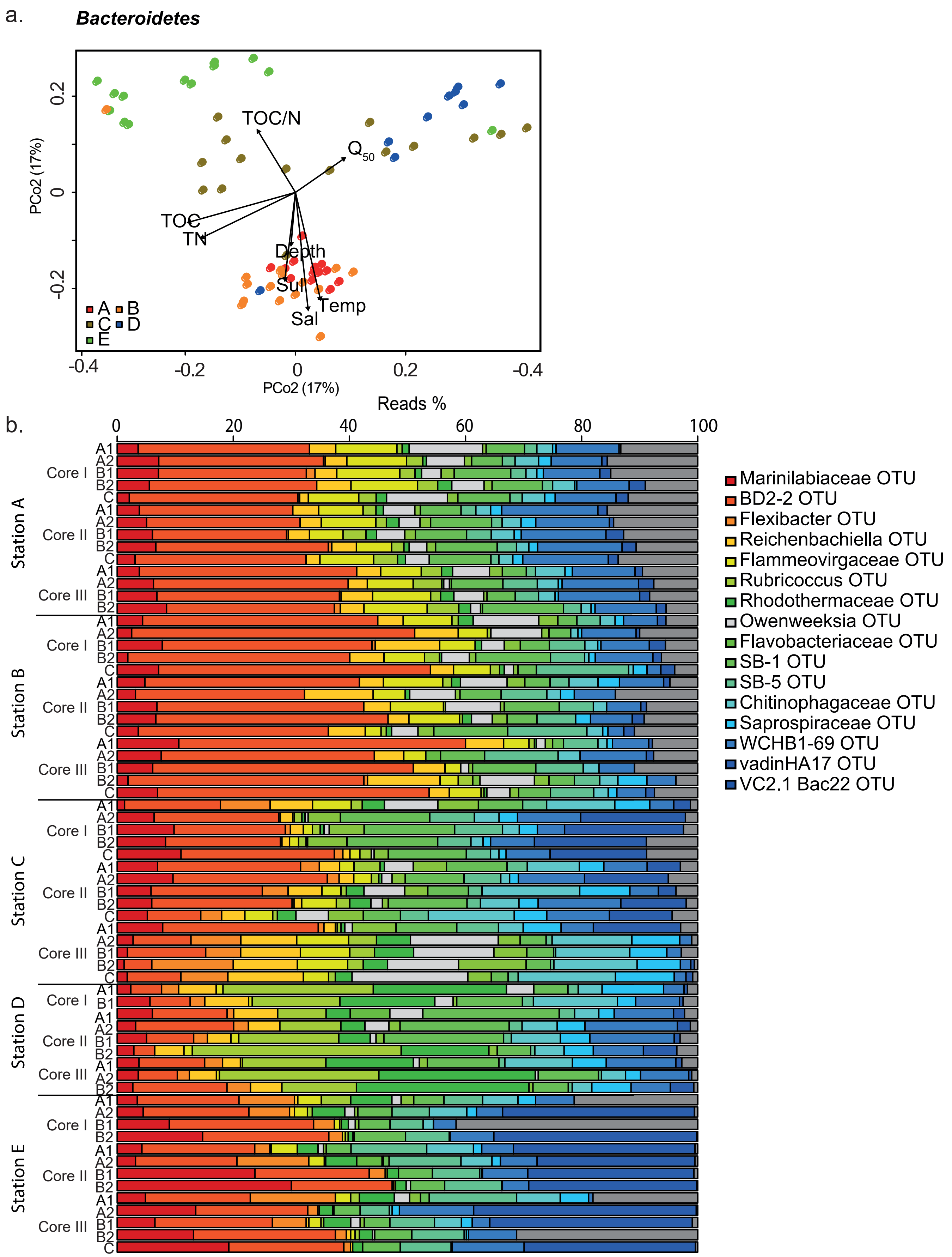

Figure S6. Analysis of the 16S rRNA assigned to the *Bacteroidetes*. (a) PCoA plot based on the Bray-Curtis dissimilarity index. (b) The abundance patterns for all OTUs assigned to *Bacteroidetes* are shown as stacked bar graphs. For clarity, the relative abundance cut-off level was 1.8% for each individual OTU assigned to the *Bacteroidetes*.

a.

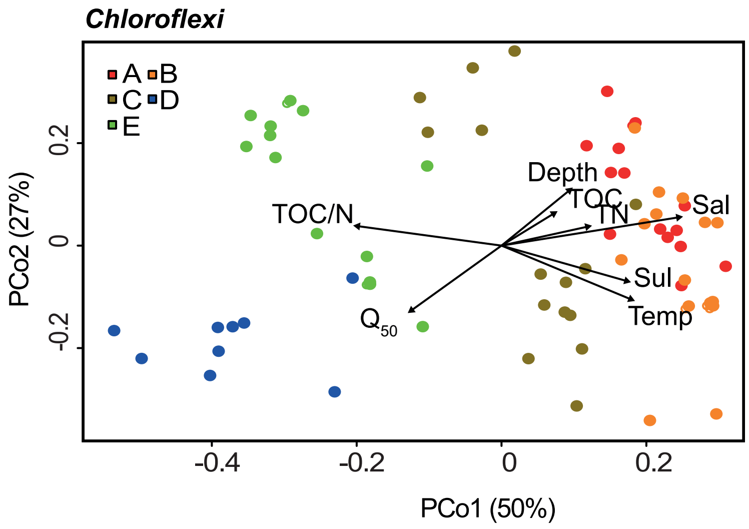

b.

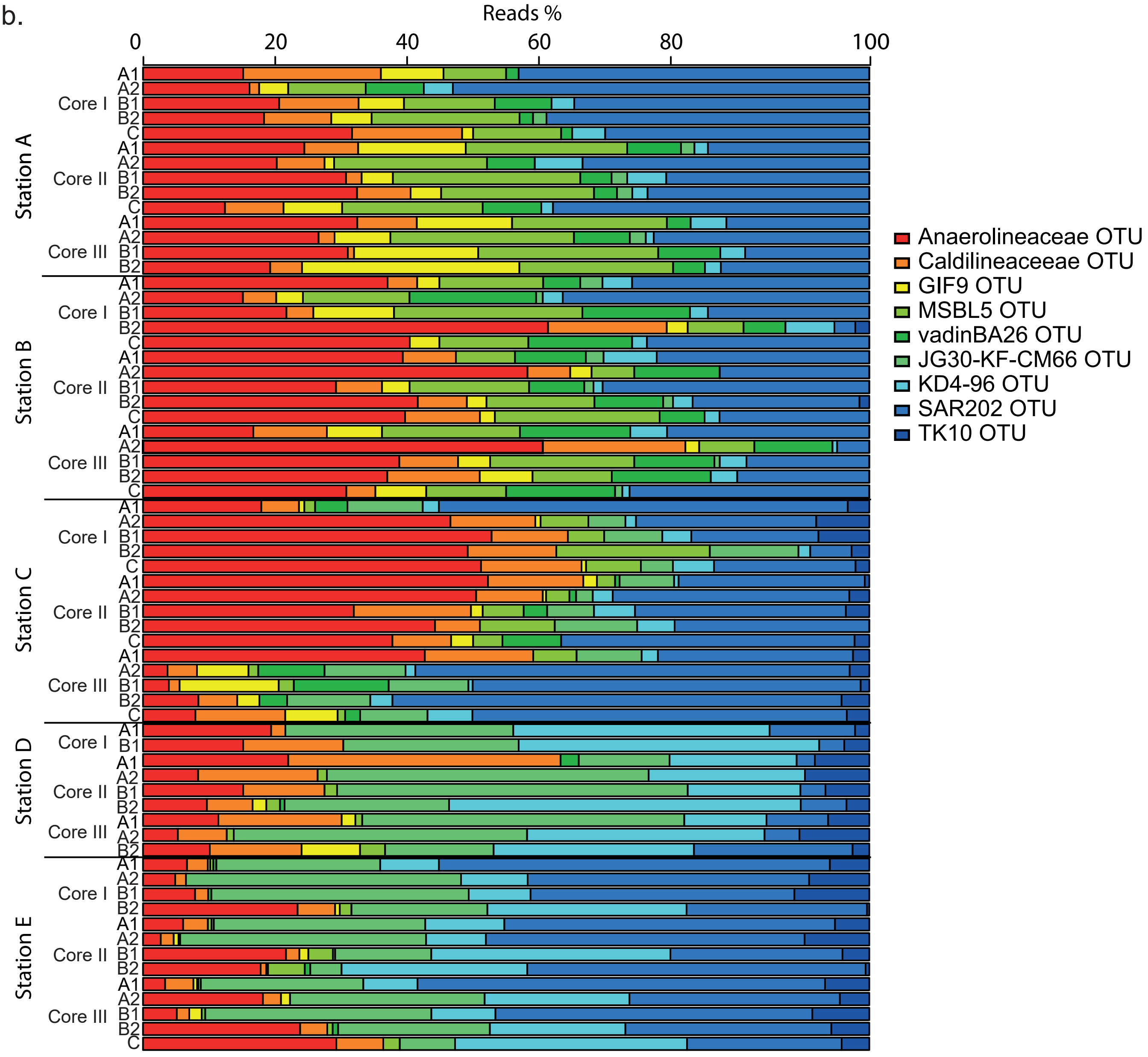

Figure S7. Analysis of the 16S rRNA assigned to the *Chloroflexi*. (a) PCoA plot based on the Bray-Curtis dissimilarity index. (b) The abundance patterns for all OTUs assigned to *Chloroflexi* are shown as stacked bar graphs. For clarity, the relative abundance cut-off level was 1.8% for each individual OTU assigned to the *Chloroflexi*.
